# Upconverting SrF_2_ nanoparticles doped with Yb^3+^/Ho^3+^, Yb^3+^/Er^3+^ and Yb^3+^/Tm^3+^ ions – optimisation of synthesis method, structural, spectroscopic and cytotoxicity studies

**DOI:** 10.1038/s41598-019-45025-1

**Published:** 2019-06-17

**Authors:** Dominika Przybylska, Anna Ekner-Grzyb, Bartosz F. Grześkowiak, Tomasz Grzyb

**Affiliations:** 1 Department of Rare Earths, Faculty of Chemistry, Adam Mickiewicz University in Poznań, Uniwersytetu Poznańskiego 8, Poznań, 61-614 Poland; 2 Department of Plant Ecophysiology, Faculty of Biology, Adam Mickiewicz University in Poznań, Uniwersytetu Poznańskiego 6, Poznań, 61-614 Poland; 3 NanoBioMedical Centre, Adam Mickiewicz University in Poznań, Wszechnicy Piastowskiej 3, Poznań, 61-614 Poland

**Keywords:** Optical materials, Nanoparticles, Cell proliferation, Biomedical materials

## Abstract

For a number of years nanomaterials have been continuously devised and comprehensively investigated because of the growing demand for them and their multifarious applications, especially in medicine. This paper reports on the properties of SrF_2_ nanoparticles (NPs) for applications in biomedicine, showing effective ways of their synthesis and luminescence under near infrared radiation - upconversion. NPs doped with lanthanide, Ln^3+^ ions (where Ln = Yb, Ho, Er, Tm) were prepared by the hydrothermal method and subjected to comprehensive studies, from determination of their structure and morphology, revealing small, 15 nm structures, through spectroscopic properties, to cytotoxicity *in vitro*. The effects of such factors as the reaction time, type and amount of precipitating compounds and complexing agents on the properties of products were characterized. The cytotoxicity of the synthesized and functionalized NPs was investigated, using human fibroblast cell line (MSU-1.1). The synthesized structures may decrease cells’ proliferation in a dose-dependent manner in the measured concentration range (up to 100 µg/mL). However, the cells remain alive according to the fluorescent assay. Moreover, the treated cells were imaged using confocal laser scanning microscopy. Cellular uptake was confirmed by the presence of upconversion luminescence in the cells.

## Introduction

Lanthanides are known as excellent for application in luminescent materials, have unique physicochemical and optical properties, being a result of 4 *f* electronic configuration. Nowadays the growing interest in research is focused on upconverting nanoparticles (UCNPs) based on host materials doped with lanthanide ions (Ln^3+^). These materials can convert low energy photons from the near-infrared range (NIR) to higher energy ones, through the multiphoton absorption process^1^. Upconverting materials can be used in various applications such as displays, solar cells, sensors, lasers, biosensors, drug delivery and many others^2–6^. Especially interesting is the application of UCNPs as biomarkers what is possible due to their excitation within biological transparency window (in the range 700–1000 nm)^4^. Thanks to this property and by using of NIR radiation, harmful effects to healthy cells are reduced in comparison to those taking place under UV excitation.

As potential biomarkers, UCNPs should exhibit high emission intensity, small size (below 50 nm) and low cytotoxicity. Their surface should also allow conjugation with biomolecules. The most prominent phosphors, which exhibit efficient emission of light under NIR radiation are based on fluoride matrices, such as, MF_2_ host materials doped with Ln^3+^ ions (where M = Ca, Sr, Ba; for more details see Table S1)^3,7–22^. These materials are characterized by low phonons energy and high chemical stability, which have direct influence on their potential applications^23,24^.

The most important step, determining NPs utilization is their effective synthesis. For this purpose, the most promising for preparing MF_2_ materials is the solvo(hydro)thermal method allowing carrying out the process under high pressure and temperature^24^. The advantages of the method are: obtaining single-phased products with small sizes of NPs, possibility of carrying out the synthesis in water, easy control of the synthesis conditions and, what is the most important, good crystallinity of products which improves their luminescence efficiency^24^.

Hydrothermal method is very common for synthesis of upconverting SrF_2_ what is reflected by many of published articles^12,14,15,23,25,26^. One of the first papers was published by J. Sun *et al*.^26^, where particles were obtained in one-step hydrothermal method with different surfactants (citric acid, EDTA, PVP, OA) to change their size and morphology. The same group of researchers obtained SrF_2_:Yb^3+^, Er^3+^ in oleate complex system in a mixture of water, ethanol and oleic acid, what allowed to obtain much smaller particles, around 5–25 nm^11^. The most known and cited method for synthesis of SrF_2_ NPs, was published by M. Pedroni *et al*.^12^. This procedure allows obtaining very small particles with a size around 8 nm and intense emission in water colloids. Based on this synthesis route, a lot of research has been done, e.g. I. Villa *et al*. obtained SrF_2_:Nd^3+^ particles for deep tissue, autofluorescence free, high resolution *in vivo* imaging using emission band at 1.340 μm^13^; M. Quintanilla *et al*. synthesized 9 nm SrF_2_:Yb^3+^,Tm^3+^ NPs in water/D_2_O colloids, with intense emission in ultraviolet range; S. Balabhada *et al*.^17^ based on SrF_2_:Yb^3+^,Er^3+^ reported a straightforward method to predict the temperature calibration curve of any upconverting thermometer based on two thermally coupled electronic levels independently of the medium. Besides, many research groups modified Pedroni’s method of SrF_2_ NPs synthesis, especially to obtain core@shell structures, e.g. S. Zanzoni *et al*.^16^ synthesized SrF_2_:Yb^3+^,Tm^3+^@SrF_2_:Yb^3+^,Er^3+^ in two-step hydrothermal synthesis for investigation of interactions between Ln-doped fluoride NPs and biomolecules whereas P. Cortelletti *et al*. published synthesis of multishell NPs^19^ used for optical thermometry. A lot of research was focused on different applications and properties of upconverting SrF_2_, (Table S1). The aqueous environment of NPs synthesis may be responsible for quenching of luminescence by –OH modes, due to non-radiative relaxation process. Therefore, design of accurate nanosized bioimaging probe requires optimization of the standard synthesis methods. Apart from the appropriate composition of the starting mixture, time and temperature of reaction, also adequate co-regents present in the synthesis medium, e.g. sodium citrate, ethylenediaminetetraacetic acid (EDTA) or cetyl trimethyl ammonium bromide (CTAB) may influence luminescence intensity of the final product and have effect on the size, shape, and degree of agglomeration of particles^3,8,12,27^. In MF_2_ doped with Ln^3+^ ions, the composition of the starting mixture may also be crucial for charge compensation within the crystal structure^12,28^.

In this paper, we present structural and spectroscopic properties of SrF_2_:Yb^3+^,Ln^3+^ NPs. Most of the published articles present spectroscopic properties of SrF_2_:Yb^3+^,Er^3+^ or just single Ln^3+^-doped NPs^9,11,13,15,26,29^. Herein we report comparison of three upconverting systems with Ho^3+^, Er^3+^ or Tm^3+^ ions as luminescence activators. NPs were prepared by hydrothermal synthesis, with two surfactants - trisodium citrate (NaCit) or ammonium citrate tribasic (NH_4_Cit) as chelating agents. Alternative synthesis procedure, for the most common, NaCit-based was developed. Also, the effects of such factors as time and amount of precipitation agent on size, the shape and agglomeration of particles, and especially spectroscopic properties were investigated. Because of potential biological applications of the synthesized NPs also their cytotoxicity was assessed. The prepared NPs were also examined as potential biomarkers. NPs may interfere with the functioning of cells, e.g. by plasma membrane integrity disruption, disturbance with organelle function or damage of the cytoskeleton^30^. The cytotoxicity depends on a lot of traits, such as size, shape, functionalization, coating, cell line used during the experiment, etc.^31^. Therefore, it is essential to assess the toxicity of newly produced NPs, which was one of the aims of the presented study. Toxicity analysis distinguishes our article from many other. Moreover, the cellular uptake of the nanostructures was imaged using confocal microscopy. Our article presents complex and wide report covering optimization of synthesis procedure, comparison of luminescence properties between products prepared in various conditions as well as with three different emitting lanthanide ions. Additionally cytotoxicity of prepared products was analysed for bare and functionalised NPs.

## Results

### Structure and morphology

**Figure 1:**
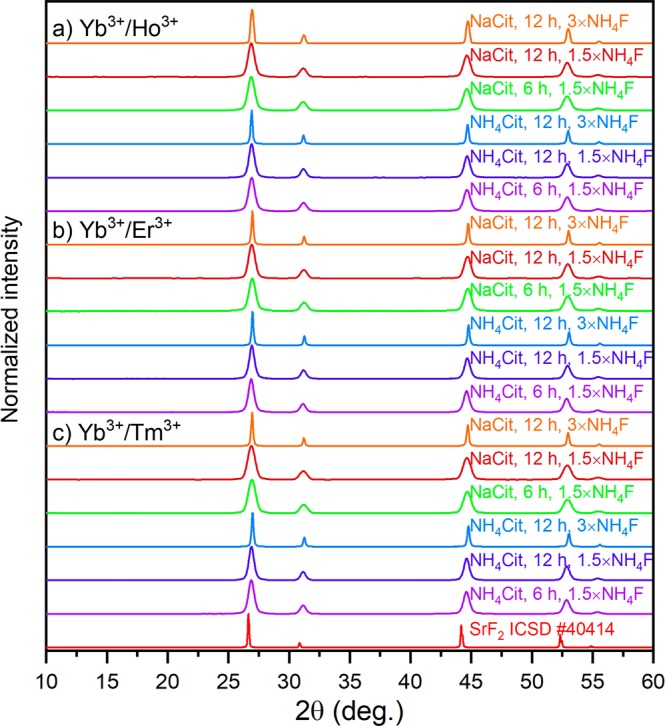
XRD patterns of the SrF_2_ samples synthesized by hydrothermal method. The patterns are described according to the scheme: source of citric ions, time of reaction, excess of NH_4_F precipitating compound (the used concentrations were: 20% Yb^3+^, 1% Ho^3+^, 1%Er^3+^ and 0.25% Tm^3+^).

Strontium fluoride doped with Ln^3+^ crystallized as cubic crystals with ${Fm}\bar{3}m$ space group (Fig. 1). Samples showed single-phased structure, however, they had different crystal sizes, which was reflected in the width of XRD peaks. NPs’ sizes determined from Scherrer equation and DLS measurements are collected in Tables S2 and S6 (for experimental results see Figs S1 and S2). Synthesis of the samples with 1.5× excess of NH_4_F within 6 and 12 h resulted in similar NPs with 11–14 nm diameter, no matter which co-regent was used. By increasing the excess of F^−^ ions to 3×, more than twice larger NPs with sizes between 28 and 39 nm were obtained. These differences in the size and morphology were caused by changes in the kinetics of the reaction and rapid growth of crystallites in the presence of higher concentration of F^−^ ions. Determined hydrodynamic diameters of the obtained NPs also confirmed the growth of their sizes from around 20–40 nm to above 100 nm when a higher amount of NH_4_F was used. The results indicate also, the tendency to agglomeration of NPs prepared with threefold excess of NH_4_F.

**Figure 2:**
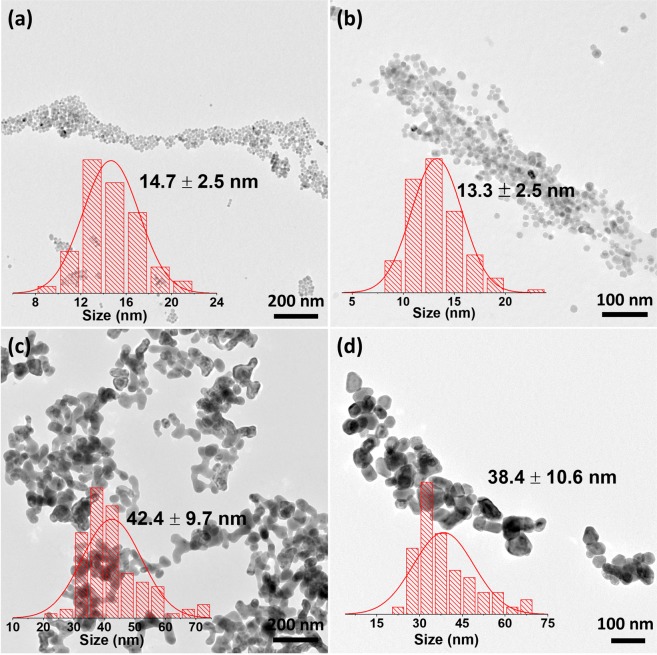
TEM images and nanocrystals size distribution of hydrothermally synthesized samples: (**a**) SrF_2_:Yb^3+^,Er^3+^, NaCit, 12 h, 1.5 × NH_4_F (**b**) SrF_2_:Yb^3+^,Er^3+^, NH_4_Cit, 12 h, 1.5 × NH_4_F (**c**) SrF_2_:Yb^3+^,Er^3+^, NaCit, 12 h, 3 × NH_4_F (**d**) SrF_2_:Yb^3+^,Er^3+^, NH_4_Cit, 12 h, 3 × NH_4_F.

TEM images show small spherical NPs with narrow size distribution when 1.5× excess of NH_4_F was used (Fig. 2a,b). The determined sizes were: 14.7 ± 2.5 nm for products obtained in the presence of NaCit as a co-reagent and 13.3 ± 2.5 nm when NH_4_Cit was used. With a 3× excess of NH_4_F much larger NPs precipitated: 42.4 ± 9.7 nm for NaCit used as a co-reagent and 38.4 ± 10.6 nm when NH_4_Cit was used. NPs prepared with higher excess of NH_4_F were of irregular shape with a wider distribution of their sizes and with visible agglomeration.

The composition of obtained products analysed by the ICP-OES technique (results in Table S3) revealed higher than expected amount of Yb^3+^ in all samples: between 21–24% when a 1.5× excess of NH_4_F was used and between 23–28% when the excess was 3×. The explanation of the extra amount of Yb^3+^ ions is complex. The smallest ionic radius of Yb^3+^ ions from all the elements used in the synthesis allowed efficient incorporation of these ions into the SrF_2_ crystal lattice (*r*Sr^2+^ = 1.26 Å, *r*Tm^3+^ = 0.9994 Å, *r*Er^3+^ = 1.004 Å, *r*Ho^3+^ = 1.0015 Å, *r*Yb^3+^ = 0.985 Å for coordination number CN = 8)^32^. High amount of charge compensating F^−^ ions presented when 3× excess of NH_4_F was added, additionally allowed embedding Yb^3+^ into the SrF_2_ structure to a higher extent. Another factor increasing the concentration of dopant ions, is the difference in solubility between SrF_2_ and LnF_3_. The latter one is less soluble in water, which favours incorporation of Ln^3+^ into the forming fluoride structure. The presence of higher amount of F^−^ ions also leads to this process (according to the solubility constant).

Elemental analysis revealed higher amount of nitrogen atoms in the samples synthesised in the presence of NH_4_Cit as co-reagent (see Table S4) in comparison with those obtained in presence of NaCit. The increased amount of nitrogen is related to NH_4_^+^ ions presented on the surface of NPs as well as ions embedded into structure of particles.

Higher concentration of Yb^3+^ in the materials resulted in reduced crystal cell volumes in comparison to those doped to a lower degree (for more see Table S5). The smallest cell volumes were calculated for the samples with 3× excess of NH_4_F and prepared with NH_4_Cit as co-reagent (for Yb^3+^/Ho^3+^, *V* = 188.09(4) Å^3^; Yb^3+^/Er^3+^ *V* = 188.10(5) Å^3^; Yb^3+^/Tm^3+^ *V* = 188.16(5) Å^3^). The reference, taken from the ICSD database, shows the cell volume for the pure SrF_2_ *V* = 194.50(7) Å^3^.

In the fluorite-type structure of MF_2_, the cations are located at the centre of a cubic unit cell, surrounded by eight F^−^ anions. The doping by Ln^3+^ replaces M^2+^ ions introducing a local charge. Charge balance may be achieved by the addition of interstitial F^−^ ions, occurring simultaneously or by introduction of M^+^ ions^12,23,28^. Along with charge compensation process, structural and spectroscopic properties of NPs may be affected. Monovalent cations (e.g. Na^+^), lead to a reduction in interatomic distance which can significantly improve upconversion properties of these materials^23,33^. In the prepared SrF_2_ NPs, there are two possibilities of charge compensation. The first, with creation of interstitial fluorine ions: Sr^2+^ → Ln^3+^ + F^−^, and the second with incorporation of monovalent cations, which were Na^+^ or NH_4_^+^ depending on the type of reagent: 2Sr^2+^ → Na^+^/NH_4_^+^ + Ln^3+ ^^28^. However, NH_4_^+^ ions are larger than Na^+^ and Sr^2+^, thus their ability to compensate charge imbalance is rather low (*r*NH_4_^+^  = 1.54 Å, *r*Na^+^  = 1.18 Å)^32^.

Zeta potentials were measured to determine the surface charge of the prepared NPs. All samples exhibited negative surface charge from −14.9 ± 5.4 mV to −33.3 ± 4.4 mV (Table S7) at physiological pH, which indicates their good stability in water as colloids and confirm the presence of negative COO^−^ groups on the surface. Furthermore, the FT-IR spectra also revealed citrate anions on the surface of the obtained particles via the presence of stretching vibrations assigned to the –CO, -CH, -COO^−^ and–OH bonds (Supplementary Materials, Fig. S3). As the result of higher amount of NH_4_^+^ ions in the materials prepared in presence of NH_4_Cit, absorption band located at around 1610 cm^−1^ revealed shift towards -NH bending range in comparison to samples prepared in the presence of NaCit.

### Spectroscopic properties

**Figure 3:**
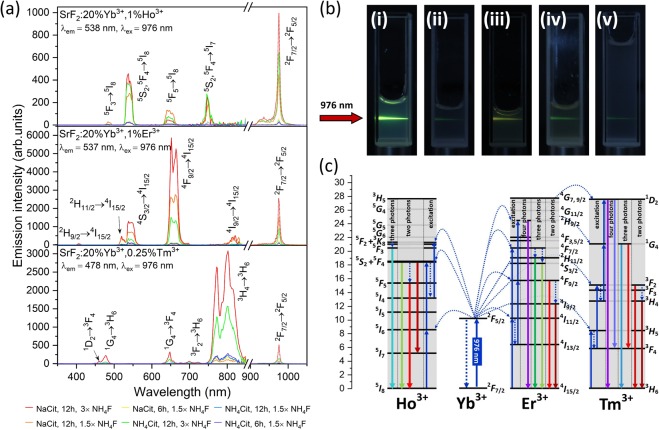
**(a)** Luminescence (300–900 nm) and excitation (>900 nm) spectra of the NPs obtained under pulsed excitation source (at 15 mJ·cm^−2^). **(b)** Emission of SrF_2_:20%Yb^3+^,1%Er^3+^ samples, where: (i) NaCit, 12 h, 3 × NH_4_F, (ii) NaCit, 12 h, 1.5 × NH_4_F, (iii) NH_4_Cit, 12 h, 3 × NH_4_F, (iv) NH_4_Cit, 12 h, 1.5 × NH_4_F, (v) NaCit, 12 h, 1.5 × NH_4_F, excited by laser (λ_ex_ = 976 nm) in water (i-iv) or phosphate-buffered saline (v) with concentration 0.1 mg/mL. **(c)** Simplified scheme of upconversion mechanism for SrF_2_: Yb^3+^, Ln^3+^ systems.

Under excitation with the NIR radiation (at 976 nm) all samples exhibited emission that resulted from the used Ho^3+^, Er^3+^ or Tm^3+^ dopant ions (Fig. 3). The brightest emissions were observed for the samples, obtained in 12 h synthesis with 3× excess of NH_4_F. The weakest emissions were recorded for the samples obtained in shorter reaction time (6 h) and with 1.5× excess of F^−^ ions.

For all samples, spectroscopic measurements revealed the emission typical of Ln^3+^ ions and energetic processes taking place in the products, such as energy transfer from Yb^3+^ to Ln^3+^ ions, excitation into higher energetic levels or emission quenching. The excitation spectra (Fig. 3a 900–1050 nm range) measured for the samples show broad and intense bands with the maximum at 976 nm, which are characteristic of the ^2^F_7/2_ → ^2^F_5/2_ transition of Yb^3+^ ions. These ions play a role of sensitisers in the studied systems, absorbing laser light. Yb^3+^ ion is perfect for this function because of its simple energy structure: one excitation energy level (^2^F_7/2_), produced by the absorption of radiation with a wavelength at around 980 nm. The intensity of this band was the highest for the samples obtained in 12 h synthesis, with 3× excess of NH_4_F, where NaCit was used as a co-reagent. The excitation band is broad in each sample, which is the effect of crystal field on the local environment of the ion resulting in Stark-splitting of the ground ^2^F_7/2_ multiplet.

The strongest emissions, similarly to the excitation spectra, were recorded for the products obtained in the 12 h synthesis with NaCit and 3× excess of NH_4_F, (see also Fig. S4). High luminescence intensity was also observed for the samples doped with Yb^3+^/Ho^3+^ and Yb^3+^/Er^3+^ obtained in 12 h synthesis with NaCit and 1.5 × NH_4_F or NH_4_Cit and 3× NH_4_F. The above results can be explained by a relatively large size of NPs, obtained during longer reaction time and with excess of fluorine ions. Small NPs are known to be prone for surface- and defects-related quenching to a greater extent than large ones^34^. Also, an increased amount of sensitiser ions (Yb^3+^), found in the samples prepared during longer synthesis (Table S5) is a factor which improved luminescence of the described structures.

The used co-reagent had a significant effect on the emission of NPs prepared. When NH_4_Cit was used, luminescence quenching was observed. This effect was caused by a larger number of NH_4_^+^ ions present on the surface of NPs in comparison to that on the products prepared in the presence of NaCit. The N-H vibrations are more efficient quenchers than O-H and our results confirm this fact.

Analysis of the ratio of intensities of the two most intense bands present in the emission spectra brings additional information about the studied systems (see Fig. 3a for the spectra or Fig. S5 for comparison of integrated emission intensities). The samples doped with a Yb^3+^/Ho^3+^ pair of ions showed the ^5^S_2_,^5^F_4_ → ^5^I_8_ band as the most intense, which resulted in green colour of emission (for CIE chromaticity diagram see Fig. S5). In the products whose emission was of lower intensity, the green (^5^S_2_,^5^F_4_ → ^5^I_8_) and red (^5^F_5_ → ^5^I_8_) emission bands had similar intensities, which was the result of decreased efficiency of excitation to the ^5^S_2_,^5^F_4_ higher excited state. Excitation into higher levels of Ln^3+^ ions usually require good crystallinity of the material and lack of quenching factors, such as NH_4_^+^ ions.

NPs doped with Yb^3+^/Er^3+^ pair ions, showed yellowish-green emission colour, as an effect of mixing of the transitions ^4^F_9/2_ → ^4^I_15/2_ (in the red range) and ^4^S_3/2_ → ^4^I_15/2_ (green range). The first one was the most intense from all observed transitions. NPs doped with Yb^3+^/Er^3+^ pair ions were characterized by the highest emission intensity from the whole studied group of compounds. The high upconversion intensity of Yb^3+^ and Er^3+^ doped samples is connected with the best match of the energies of the excited levels of the used activator ions (Yb^3+ 4^F_5/2_ → Er^3+ 4^I_11/2_), thanks to which the energy transfer between these ions is more efficient. Depending on the synthesis conditions, the ratio between red and green transition bands was slightly different, revealing the influence of NPs’ sizes and charge compensation issues on the UC mechanism and quenching processes efficiency (see Figs S5 and S6). Increased red emission band is usually connected with non-radiative relaxation form the ^4^S_3/2_ excited state what in the presented results is an effect of NH_4_^+^ ions presence and lower crystallinity of the products obtained at shorter time and with smaller NPs’ sizes.

The samples doped with Yb^3+^/Tm^3+^ pairs of ions showed blue or pink-blue UC with the domination of ^3^H_4_ → ^3^H_6_ transition (NIR band) in the spectra (Figs 3a, S5 and S6). The ratio between ^1^G_4_ → ^3^H_6_ and ^3^H_4_ → ^3^H_6_ transitions was similar for each type of co-reagent used. However, for the systems in which quenching through to the presence of –NH oscillators was noticeable in the emission intensity, the colour of emission was slightly shifted to the red (Fig. S6). The ^1^D_2_ and ^1^G_4_ excited states of Tm^3+^ are more sensitive to the quenching factors as evidenced by a change in the emission colour. The observed spectroscopic properties of SrF_2_:Yb^3+^,Tm^3+^ NPs shows that colour of luminescence may be controlled during the synthesis, by co-reagents affecting the quenching processes.

Figure 3b shows the luminescence of SrF_2_:20%Yb^3+^,1%Er^3+^ NPs in the form of water colloids under excitation with 976 nm laser light. The intense emission of water colloids allow a description of prepared NPs as potentially useful for biomedical applications especially for bioimaging as we show below. A scheme of the upconversion mechanism responsible for the observed emissions is presented in Fig. 3c.

On the basis of luminescence decays measured for the obtained product (Supplementary Materials, Fig. S7), the luminescence lifetimes were calculated and are collected in Table 1. The values of lifetimes for the Yb^3+^/Ho^3+^ doped samples are strongly related to the luminescence intensity. The longest lifetimes (147 μs for the ^5^F_5_ → ^5^I_8_ and 118 μs for ^5^S_2_, ^5^F_4_ → ^5^I_8_ transition) were determined for the best emitting sample prepared in 12 h synthesis, with NaCit as co-reagent and 3× excess of NH_4_F. An analogous sample, but prepared in the presence of NH_4_Cit, had a similar value of lifetime. Luminescence lifetimes determined for the ^5^F_5_ → ^5^I_8_ transition, which occurred in the red spectral range, were usually the longest ones, which is related to the necessity of non-radiative transition between the ^5^S_2_, ^5^F_4_ and ^5^F_5_, excited states. The shortest decay time was measured for the sample obtained in 6 h synthesis and with NaCit as co-reagent. The reason is very weak luminescence of this highly quenched sample.

**Table 1:** Emission lifetimes calculated on the basis of the measured luminescence decays of SrF_2_:Yb^3+^,Ln^3+^ NPs under 976 nm laser excitation (for decays see Fig. S8, err < 0.1 μs).

Co-reagent	NH_4_F excess	Reaction time (h)	Lifetime (μs)											
			SrF_2_:20%Yb^3+^,1%Ho^3+^				SrF_2_:20%Yb^3+^,1%Er^3+^				SrF_2_:20%Yb^3+^,0.25%Tm^3+^			
			^5^F_3_ → ^5^I_8_	^5^S_2,_^5^F_4_ → ^5^I_8_	^5^F_5_ → ^5^I_8_	^5^S_2,_^5^F_4_ → ^5^I_7_	^2^H_9/2_ → ^4^I_15/2_	^2^H_11/2_ → ^4^I_15/2_	^4^S_3/2_ → ^4^I_15/2_	^4^F_9/2_ → ^4^I_15/2_	^1^D_2_ → ^3^F_4_	^1^G_4_ → ^3^H_6_	^1^G_4_ → ^3^F_4_	^3^H_4_ → ^3^H_6_
**NaCit**	**1.5×**	**6**	13.5	21.0	29.2	16.8	16.9	25.4	49.3	48.7	118.3	374.0	364.8	214.0
		**12**	15.7	27.3	35.0	24.0	30.3	33.4	33.5	108.6	204.2	425.8	420.6	284.2
	**3×**	**12**	51.5	117.8	146.5	103.9	74.4	82.1	90.6	235.7	309.3	596.6	592.3	492.2
**NH**_**4**_**Cit**	**1.5×**	**6**	17.6	22.1	39.5	24.4	29.8	41.2	37.9	89.4	145.8	341.9	352.8	263.5
		**12**	11.4	31.6	37.1	24.1	26.0	29.7	30.4	97.0	147.0	357.0	366.3	252.7
	**3×**	**12**	44.4	96.7	110.8	81.3	46.2	55.2	63.7	174.4	248.6	511.7	500.5	368.6

Analysis of luminescence decays measured for the samples doped with Yb^3+^/Er^3+^ ions, revealed that the longest lifetimes were typical of products obtained in 12 h synthesis in the presence of NaCit and 3× excess of NH_4_F, similarly to the Yb^3+^/Ho^3+^ doped samples. The lifetime assigned to the ^4^F_9/2_ → ^4^I_15/2_ transition was 236 μs for the sample prepared in the presence of NaCit and 174 μs when NH_4_Cit was used as co-reagent. Lifetimes were slightly shorter for the compounds prepared with NH_4_Cit as a chelating agent, because of the earlier mentioned quenching effect of -NH modes. The longest lifetimes calculated for the red band can be related to the Er^3+^ excitation mechanism to the ^4^F_9/2_ level. There are two possibilities of the excitation: one connected with non-radiative transition from ^2^H_11/2_ level and the other with non-radiative transition from ^4^I_11/2_ to ^4^I_13/2_ excited state followed by absorption of a photon to ^4^F_9/2_ level (^4^I_13/2_ (Er^3+^) + ^2^F_5/2_ (Yb^3+^) → ^4^F_9/2_ (Er^3+^) + ^2^F_7/2_ (Yb^3+^)). The first excitation pathway occurs when the emission lifetime of ^4^F_9/2_ → ^4^I_15/2_ transition is similar to that of ^4^S_3/2_ → ^4^I_15/2_, which is observed for the compound synthesized in a 6 h process with 1.5× excess of NH_4_F and in the presence of NaCit. The second mechanism can explain the observed spectroscopic properties if the decay time of the transition ^4^F_9/2_ → ^4^I_15/2_ is twice as long (or even more) as the ^4^S_3/2_ → ^4^I_15/2_ transition lifetime. That is true for all samples except the one mentioned above. If the first pathway of the ^4^F_9/2_ population dominates, the decay time of ^4^F_9/2_ level should be similar to that of ^4^S_3/2_. But if the energy transfer prevails, the decay time of the ^4^F_9/2_ level depends on the decay times of ^4^I_13/2_ (Er^3+^) and ^2^F_5/2_ (Yb^3+^).

The SrF_2_:Yb^3+^,Tm^3+^ NPs showed the longest decays from all obtained samples. Furthermore, similarly as for the samples doped with Yb^3+^/Ho^3+^ or Yb^3+^/Er^3+^ ions described above, the sample prepared in 12 h synthesis, with 3 × NH_4_F and with the use of NaCit, was characterized by the highest value of emission lifetime (596.6 μs for ^1^G_4_ → ^3^H_6_ and 592.3 μs ^1^G_4_ → ^3^F_4_). Similarly to the samples with Yb^3+^/Ho^3+^ and Yb^3+^/Er^3+^, those prepared with NH_4_Cit as a complexing agent showed shorter lifetimes in comparison to that of the analogous product prepared with NaCit. Decay times strongly depended on the population mechanism of the excited states. Also, quenching factors present in the system affected the determined emission lifetimes.

For better understanding of the upconversion mechanism, dependencies of the luminescence intensity on laser powers were measured. The relation between the UC intensity *I* and the pumping excitation power density, *P* is given by the equation^35^:

$$I\propto {P}^{n}$$

where *n* is the number of photons required to populate the excited state. The results of the calculations are collected in Table 2 (see Fig. S8 for the experimental data).

**Table 2:** Number of photons involved in the upconversion mechanism, determined from the dependencies of luminescence intensity on laser power for SrF_2_:Yb^3+^,Ln^3+^ NPs (for experimental results see Fig. S9, err < 0.15).

Co-reagent	NH_4_F excess	Reaction time (h)	Number of photons									
			SrF_2_:20%Yb^3+^,1%Ho^3+^			SrF_2_:20%Yb^3+^,1%Er^3+^			SrF_2_:20%Yb^3+^,0.25Tm^3+^			
			^5^S_2,_^5^F_4_ → ^5^I_8_	^5^F_5_ → ^5^I_8_	^5^S_2,_^5^F_4_ → ^5^I_7_	^2^H_11/2_ → ^4^I_15/2_	^4^S_3/2_ → ^4^I_15/2_	^4^F_9/2_ → ^4^I_15/2_	^1^D_2_ → ^3^F_4_	^1^G_4_ → ^3^H_6_	^1^G_4_ → ^3^F_4_	^3^H_4_ → ^3^H_6_
**NaCit**	**1.5×**	**6**	1.9	2.0	1.0	1.7	1.8	1.7	2.4	2.5	1.3	1.9
		**12**	2.0	2.2	1.5	1.7	1.8	1.8	1.8	2.1	2.0	1.7
	**3×**	**12**	1.8	1.8	1.5	1.8	1.6	1.7	1.2	1.1	2.1	1.2
**NH**_**4**_**Cit**	**1.5×**	**6**	0.7	1.6	0.7	0.9	1.1	1.2	0.8	1.6	1.9	1.7
		**12**	1.0	1.2	0.8	1.0	1.1	1.3	1.3	1.0	1.5	1.3
	**3×**	**12**	1.8	1.9	1.6	0.8	0.8	1.0	1.3	1.3	1.3	1.2

To achieve ^5^S_2_/^5^F_4_ or ^5^F_5_ excited state of Ho^3+^ ions two photons are required. The result of experiments, shown in Table 2, are in some cases different from the theoretical ones. For the samples prepared in 6 h synthesis and with NaCit as well as with NH_4_Cit as co-reagents, the number of photons was lower than expected, which was caused by non-radiative relaxation to the ^5^F_5_ lower excited state. The highest slope value was obtained for the sample prepared with 3× excess of NH_4_F, which is also connected with intense emission and long lifetimes determined for this sample. The slope coefficients determined for NPs doped with a Yb^3+^/Er^3+^ pair of ions are higher than those for almost all samples (except the samples prepared in the presence of NH_4_Cit), which confirms the two-photon excitation process. For the samples doped with Yb^3+^/Tm^3+^ ions, the dependencies of the emission intensity on laser energies were measured for four transitions. According to the scheme in Fig. 3c, the ^1^D_2_ energy level requires four photons in the excitation process, ^1^G_4_ three photons, and ^3^H_4_ two photons. From the experimental data only for the ^3^H_4_ → ^3^H_6_ transition (two-photon process), the number of photons was close to the theoretical value.

Summarising, for almost all samples the slope coefficient values are lower than expected, especially for Yb^3+^/Tm^3+^ dopants. There are many factors which can affect the UC process and lower slope values, e.g. saturation effect, heating of samples or cross-relaxation process between dopants. Nanomaterials are particularly prone to quenching processes, and their crystal structure is often defected which yields luminescence deactivation centres.

On the basis of the collected and literature data, the UC mechanism proposed for SrF_2_:Yb^3+^,Ln^3+^ NPs is presented in Fig. 3c. In these systems, Yb^3+^ ions absorb photons, which results in excitation of the Yb^3+^ ion from ^2^F_7/2_ to ^2^F_5/2_ excited state. In the next step, the energy may be dissipated, leading to Yb^3+^ ion in its ground state or, as a result of energy transfer upconversion (ETU), to the Ln^3+^ ion (^5^I_6_ energy level for Ho^3+^, ^4^I_11/2_ for Er^3+^ and ^3^H_5_ for Tm^3+^). To achieve an appropriate energy level for Ho^3+^ and Er^3+^ in the samples, the two-photon process is required. For Tm^3+^ from two to four photons must be transferred.

### Biological properties

**Figure 4:**
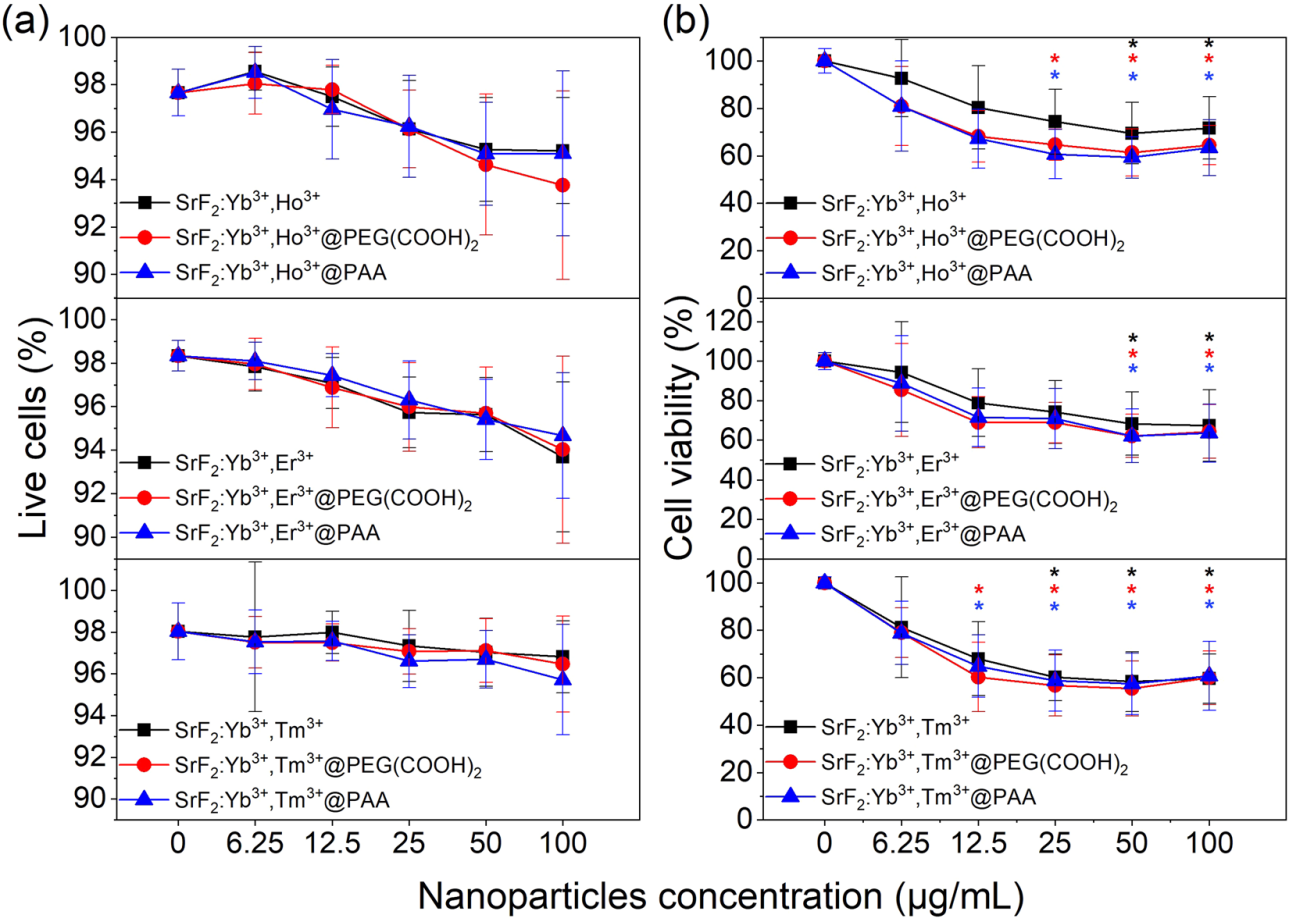
**(a)** Influence of SrF_2_:Yb^3+^,Ln^3+^, SrF_2_:Yb^3+^,Ln^3+^@PEG(COOH)_2_, SrF_2_:Yb^3+^,Ln^3+^@PAA on human fibroblasts’ viability, determined by the Live/Dead cell viability assay. The data are shown as mean values with the standard deviation (means ± SD). **(b**) Influence of SrF_2_:Yb^3+^,Ln^3+^, SrF_2_:Yb^3+^,Ln^3+^@PEG(COOH)_2_, SrF_2_:Yb^3+^,Ln^3+^@PAA on human fibroblasts’ proliferation, determined by WST assay. Asterisks indicate results significantly different from those obtained for the control (Kruskal-Wallis test, significance at p < 0.05). The data are shown as mean values with the standard deviation (means ± SD).

**Figure 5:**
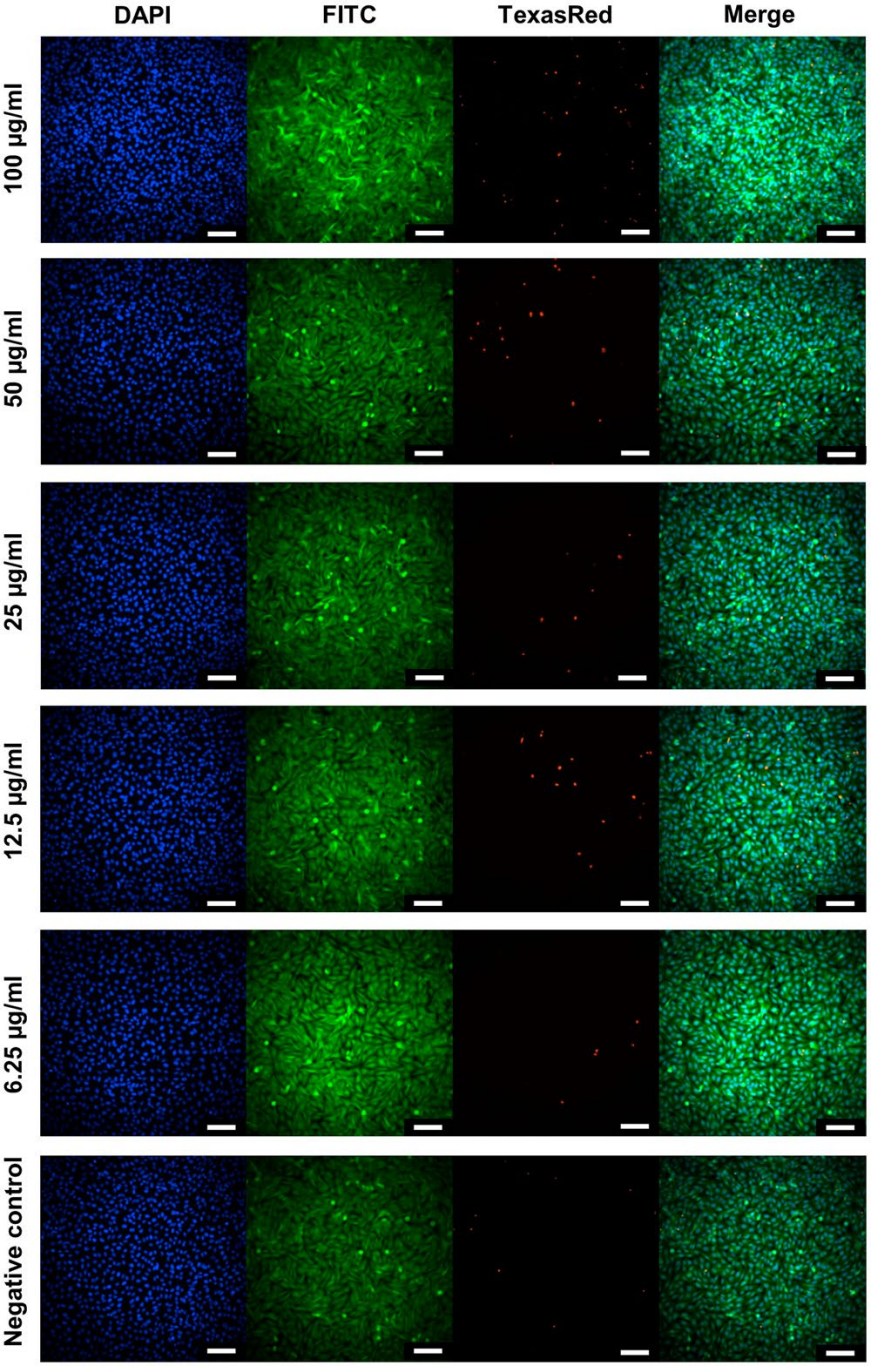
Representative high-content images of MSU1.1 cells exposed to SrF_2_:20%Yb^3+^,0.25%Tm^3+^ nanoparticles (6.25–100 µg/mL). Images were obtained using different filters to detect nuclei (DAPI), live cells (FITC), and dead cells (TexasRed). The scale bars denote 100 µm.

Cell health and growth can be determined by quantifying different parameters. In the presented study, the viability of cells treated with the studied NPs was investigated using the WST-1 and Live/Dead cell viability assays (see Supplementary Materials for more details). The WST-1 assay determines the cell metabolic activity and proliferation, whereas the Live/Dead assay determines the ratio of alive to dead cells in the population. Fluorescent Live/Dead cell viability assay showed that the proportions of alive and dead cells incubated with NPs were similar to the control cells (Fig. 4a). Representative images of the cells exposed to the analysed NPs are displayed in Fig. 5 and Figs S9–11. However, the WST-1 test showed contrary results. Namely, NPs caused a significant decrease in the proliferation rate in a dose-dependent manner in the measured concentration range (up to 100 µg/mL) (Fig. 4b). Moreover, the cytotoxic effect differs between the NPs doped with various ions. From among bare NPs (not coated with PEG–(COOH)_2_ or PAA) the structures doped with Tm^3+^ were more cytotoxic than those doped with Ho^3+^ and Er^3+^. Namely, the cells treated with SrF_2_:20%Yb^3+^,0.25%Tm^3+^ at concentration 25 µg/mL showed significantly lower proliferation rate than the control cells, whereas the other two bare nanostructures showed a negative influence on the cells at 50 µg/mL. The obtained results are in contrast to some of the earlier reports claiming that the synthesized fluorides doped with Ln^3+^ ions had no or low impact on mammalian cells^36,37^. However, other authors reported that some of the NPs may decrease cell viability even at relatively low concentrations^38^.

To improve the bio-application and decrease the cytotoxicity NPs are usually coated and functionalized as described previously^31,39^. These procedures were also generally used to improve other properties of nanostructures, such as dispersion ability and stability of NPs in aqueous solutions, luminescence properties, shape or protection from the surrounding environment. The studied NPs were coated with two different organic compounds, namely PEG–(COOH)_2_ and PAA. In the case of NPs doped with Ho^3+^ their modification with the PEG–(COOH)_2_ and PAA caused a slight increase of the cytotoxicity (Fig. 4b). This effect was not observed when the cells were incubated with SrF_2_:20%Yb^3+^,1%Er^3+^ and SrF_2_:20%Yb^3+^,0.25%Tm^3+^. The obtained results are in accordance with those Das and co-authors, who claim that PEG-oleate capped NaYF_4_:Yb^3+^,Er^3+^ significantly decrease the viability of Human Aortic Endothelial Cells in comparison to bare NPs^40^, although in most of the previous research, NPs coated with PEG–(COOH)_2_ and PAA were considered as a less toxic than bare NPs^38,39^.

**Figure 6:**
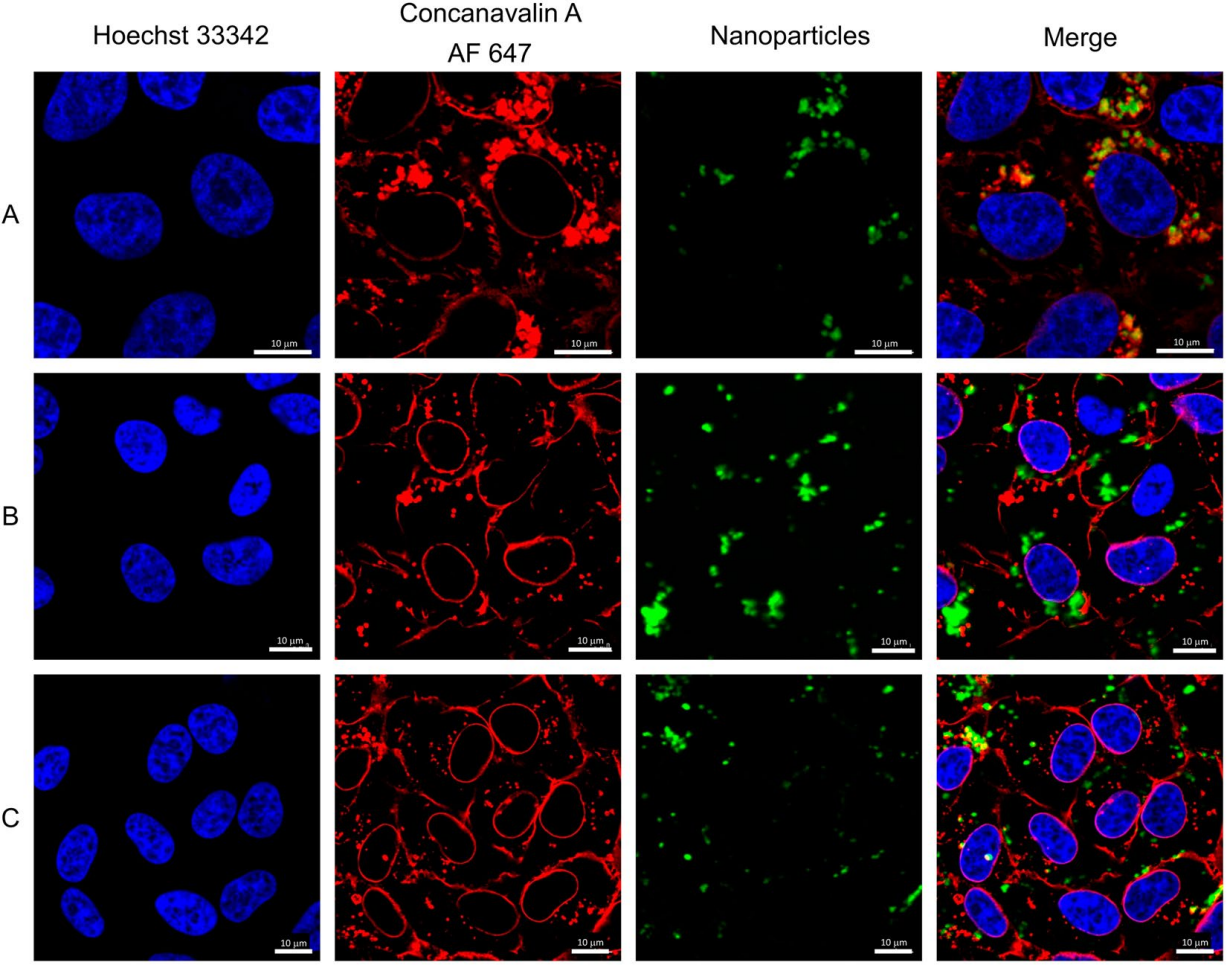
Human fibroblasts after 24 h incubation with the: (**A**) SrF_2_:20%Yb^3+^,0.25%Tm^3+^, (**B**) SrF_2_:20%Yb^3+^0.25%Tm^3+^@PEG(COOH)_2_, (**C**) SrF_2_:20%Yb^3+^0.25%Tm^3+^@PAA, imaged using confocal laser scanning microscopy equipped with a tuneable infrared laser. Red colour - cell membrane (concanavalin 647, exc. 633 nm), blue colour – cell nuclei (DAPI, exc. 405 nm), green colour – NPs (NPs’ luminescence, infrared excitation).

The confocal microscopy study demonstrated that all of the synthetized NPs were easily internalized by the fibroblast cells (Fig. 6 and Figs S12, S13), despite their negative charge, as confirmed by the presence of upconversion luminescence. High cellular uptake of negatively charged NPs results from strong and nonspecific interactions with the plasma membrane^41^. The results are in accordance with previous research, showing that upconverting fluorides doped with Ln^3+^ ions are suitable for imaging^42,43^.

## Discussion

SrF_2_:Yb^3+^,Ln^3+^ nanoparticles (where Ln = Ho, Er, Tm) can be synthesised by hydrothermal method using metal salts and NH_4_F as reagents, NaCit or NH_4_Cit used as co-reagents allowing for control reaction kinetics. The influence of complexation agents, as well as reaction time and amount of ammonium fluoride on morphology and spectroscopic properties, was studied in details. The size of obtained NPs was slightly depended on the reaction time, which was 6 or 12 h. Much more important was the concentration of NH_4_F precipitating reagent. Two concentrations were used: 1.5× and 3× excess to the stoichiometric amount. Lower concertation of NH_4_F resulted in NPs with sizes around 11–15 nm, however, higher excess caused precipitation of slightly agglomerated NPs with sizes between 28–39 nm. The used synthesis conditions also affected dopants concentrations what was especially visible in case of Yb^3+^ ions. Smaller particles, prepared in the presence of 1.5× excess of NH_4_F, usually had between 20–23% mol. of Yb^3+^ ions, where those prepared with 3× excess of NH_4_F 23–28% mol. The applied conditions also had the influence of hydrodynamic diameter of NPs in water colloids. 1.5× excess of NH_4_F resulted in NPs with diameter around 18–47 nm, whereas 3× excess in the formation of larger clusters with diameter 100–250 nm, most probably consisted of several NPs. The prepared NPs were negatively charged at physiological pH, what was a result of the presence of citrate groups on their surface. Determined zeta potentials were in the range of −15 to −33 mV.

The applied synthesis conditions had a great influence on the spectroscopic properties of obtained NPs. The most intense emission under NIR wavelengths was obtained for products prepared in 12 h synthesis, with 3 × NH_4_F excess and NaCit as a complexing agent. Furthermore, reaction time and added co-reagent had also effect on the upconversion colour, what was a result of changes in the intensity of emission bands. Measured luminescence decays as well as dependences of luminescence intensity on laser powers confirmed energy transfer between Yb^3+^ and Ho^3+^, Er^3+^ or Tm^3+^ as process responsible for observed upconversion.

Summarising, high upconversion intensity can be achieved only if a large excess of NH_4_F reagent is used, the reaction time is long (12 h) and as co-reagent, NaCit is used. On the other hand, small NPs with narrow size distribution can be obtained when reaction time is short (6 h) and NH_4_F precipitating agent is at low concentration.

The cytotoxicity of the synthesized and functionalized NPs was investigated, using WST-1 cell proliferation assay and Live/Dead cell viability assay. We demonstrated that the synthesized structures exhibited proliferative inhibition in fibroblast cells in a dose-dependent manner, whereas they appeared to be alive according to fluorescent assay. Further *in vitro* toxicity evaluation with the aim to discover the mechanisms of impact of NPs on the cells has to be performed. Cellular uptake of the NPs was confirmed by the presence of upconversion luminescence in the cells. The luminescent properties of the nanoparticles allow them to be used as fluorescent agents in bio-imaging applications.

## Methods

### Materials

Rare earth (RE) oxides: Er_2_O_3_ and Yb_2_O_3_ (99.99%, Stanford Materials, United States) were dissolved separately in hydrochloric acid, HCl (ultra-pure, Sigma-Aldrich, 37%, Poland) in order to obtain respective rare earth chloride solutions in a concentration of 1 or 0.25 M. Ammonium fluoride, NH_4_F (98+%, Sigma-Aldrich, Poland) was used as the source of fluoride ions. Strontium chloride hexahydrate SrCl_2_·6H_2_O (Sigma-Aldrich, 99+%, Poland), citric acid trisodium salt dihydrate, (Sigma-Aldrich, 97%, Poland) and ammonium citrate tribasic, (Sigma-Aldrich, ≥97%, Poland), phosphate buffered saline (BioShop), poly(ethylene glycol) bis(carboxymethyl) ether (average M_n_ 250, Sigma-Aldrich, Poland), polyacrylic acid (average M_w_ 1800, Sigma – Aldrich, Poland) were used as received, without further purification. Deionized water was used for the synthesis.

### Synthesis of SrF_2_:Ln^3+^ nanoparticles

To obtain 3.5 mmol of SrF_2_ doped with 20% of Yb^3+^ and 1% of Ho^3+^, 2.77 mL of 1 M SrCl_2_ solution and YbCl_3_ mixed with HoCl_3_ (0.70 mL of 1 M YbCl_3_ and 0.14 mL of 0.25 M HoCl_3_) were added to 20 mL of 1 M sodium citrate, NaCit solution (anti-agglomeration and complexing agent) or 1 M solution of ammonium citrate, NH_4_Cit. Then, 5 mL of 2.10 M or 4.20 M solution of NH_4_F (depending on the NH_4_F excess) were added to the solution containing SrCl_2_ and LnCl_3_ salts. The pH of the final mixture was equal to 7.5. The as-prepared transparent solution was transferred into the 50 mL Teflon-lined vessel and hydrothermally treated for 6 h or 12 h (180 °C, 15 bars), in an externally heated autoclave (Berghof DAB-2). When the reaction was completed, the obtained white precipitate was purified by centrifugation and rinsed several times with water and ethanol. The final product was dried under ambient conditions. NPs doped with Er^3+^ or Tm^3+^ were prepared analogously.

### Surface functionalization of SrF_2_:Yb^3+^,Ln^3+^ nanoparticles

Three samples: SrF_2_:20%Yb^3+^,1%Ho^3+^, SrF_2_:20%Yb^3+^,1%Er^3+^ and SrF_2_:20%Yb^3+^,0.25%Tm^3+^ prepared by 12 h synthesis in the presence of NaCit and with 1.5× excess of NH_4_F were selected for cytotoxicity studies. The samples showed necessary parameters for bioimaging such as good spectroscopic properties and small NPs’ sizes, around 15 nm.

To modify surface of NPs, 100 mg of each sample was dissolved in 30 mL of phosphate-buffered saline (PBS). After that, the solution was ultrasonicated for 1 h. Next, 4 mL of each NPs solution was mixed with 4 mL of 1% solution of poly(ethylene glycol) bis(carboxymethyl) ether (PEG–(COOH)_2_) in PBS or 4 mL of 1% solution of polyacrylic acid (PAA) in PBS. After another 0.5 h of sonification, samples where centrifuged, washed with PBS solution three times, and dissolved in PBS. As prepared colloids were diluted by PBS solution to receive 100 µg/mL concertation of NPs.

### Characterization

Powder diffractograms were recorded on a Bruker AXS D8 Advance diffractometer in the Bragg-Brentano geometry, with Cu K_*α*1_ radiation λ = 1.5406 Å, in the 2*θ* range from 6 to 60°. The reference data was taken from the Inorganic Crystal Structure Database (ICSD). The composition of prepared materials was analysed by Inductively Coupled Plasma-Optical Emission Spectrometer Varian ICP-OES VISTA-MPX and EA Vario EL III. Transmission-electron-microscopy (TEM) images were recorded on an FEI Tecnai G2 20 X-TWIN transmission electron microscope, which used an accelerating voltage of 200 kV. Fourier transform infrared spectra (FT-IR) were recorded using a JASCO 4200 FT-IR spectrophotometer. DLS and zeta potential measurements were performed by using a Malvern Zetasizer Nano ZS instrument.

The luminescence characteristics (excitation, emission spectra, luminescence decays) of the prepared samples were measured on a QuantaMaster^TM^ 40 spectrophotometer equipped with an Opolette 355LD UVDM tunable laser, with a repetition rate of 20 Hz and a Hamamatsu R928 photomultiplier used as a detector for emission/excitation spectra and decay time measurements. A continuous Dragon Lasers DPSS 980 nm laser was used as the excitation source, coupled to a 200 µm optical fibre and collimator to determine dependencies between emission intensity and laser power.

### Cytotoxicity

#### Cell culture

Human fibroblast (MSU-1.1) cell line from Prof. C. Kieda (CBM, CNRS, Orleans, France), was cultured in Dulbecco’s modified Eagle’s medium (DMEM, Gibco), supplemented with 10% fetal bovine serum (FBS, Sigma-Aldrich) and 1% penicillin-streptomycin antibiotics (Sigma-Aldrich). The cells were maintained at 37 °C in a humidified atmosphere supplemented with 5% CO_2_. The cell morphology was checked using an inverted microscope (Leica DMIL LED).

#### Cytotoxicity assays

For the cytotoxicity test, 5·10^3^ cells/well were seeded at 96-well plate and incubated for 24 h. Afterwards, 50 µl of several different concentrations of the NPs diluted in PBS were added to 150 µl of culture medium in each well resulting in a final concentration of 100, 50, 25, 12.5 and 6.25 µg/mL, and the cells were further incubated for 48 h. Phosphate buffered saline (PBS, Sigma-Aldrich) was used as a control. For more details see Supporting Information.

## Supplementary information

Supporting Information
