# Author Correction: Upconverting SrF_2_ nanoparticles doped with Yb^3+^/Ho^3+^, Yb^3+^/Er^3+^ and Yb^3+^/Tm^3+^ ions – optimisation of synthesis method, structural, spectroscopic and cytotoxicity studies

**DOI:** 10.1038/s41598-019-53897-6

**Published:** 2019-11-21

**Authors:** Dominika Przybylska, Anna Ekner-Grzyb, Bartosz F. Grześkowiak, Tomasz Grzyb

**Affiliations:** 1 Department of Rare Earths, Faculty of Chemistry, Adam Mickiewicz University in Poznań, Uniwersytetu Poznańskiego 8, Poznań, 61-614 Poland; 2 Department of Plant Ecophysiology, Faculty of Biology, Adam Mickiewicz University in Poznań, Uniwersytetu Poznańskiego 6, Poznań, 61-614 Poland; 3 NanoBioMedical Centre, Adam Mickiewicz University in Poznań, Wszechnicy Piastowskiej 3, Poznań, 61-614 Poland

Correction to: *Scientific Reports* 10.1038/s41598-019-45025-1, published online 17 June 2019

In the original version of this Article, Anna Ekner-Grzyb was incorrectly affiliated with ‘Faculty of Biology, Adam Mickiewicz University in Poznań, Uniwersytetu Poznańskiego 6, Poznań, 61-614, Poland’. The correct affiliation is listed below.

Department of Plant Ecophysiology, Faculty of Biology, Adam Mickiewicz University in Poznań, Uniwersytetu Poznańskiego 6, Poznań, 61-614, Poland

This error has now been corrected in the PDF and HTML versions of the Article.

