# Supplementary material for: Upconverting SrF2 nanoparticles doped with Yb3+/Ho3+, Yb3+/Er3+ and Yb3+/Tm3+ ions – optimisation of synthesis method, structural, spectroscopic and cytotoxicity studies
Source: Sci Rep. 2019 Jun 17;9:8669. doi: 10.1038/s41598-019-45025-1 (PMC6572793; doi:10.1038/s41598-019-45025-1)
Supplement: Supplementary file 1 — Supporting Information [file 41598_2019_45025_MOESM1_ESM.pdf]

Upconverting SrF_2 nanoparticles doped with Yb^{3+}/Ho^{3+}, Yb^{3+}/Er^{3+} and Yb^{3+}/Tm^{3+} ions – optimisation of synthesis method, structural, spectroscopic and cytotoxicity studies

Dominika Przybylska[1], Anna Ekner-Grzyb[2], Bartosz F. Grześkowiak[3], and Tomasz Grzyb[1,*]

[1]Department of Rare Earths, Faculty of Chemistry, Adam Mickiewicz University in Poznań, Uniwersytetu Poznańskiego 8, Poznań, 61-614, Poland
[2]Department of Plant Ecophysiology, Faculty of Biology, Adam Mickiewicz University in Poznań, Uniwersytetu Poznańskiego 6, Poznań, 61-614, Poland
[3]NanoBioMedical Centre, Adam Mickiewicz University in Poznań, Wszechnicy Piastowskiej 3, Poznań, 61-614, Poland

*E-mail: tgrzyb@amu.edu.pl

Supplementary Materials

Introduction

Table S1 shows review of literature about SrF_2 upconverting nanoparticles with basic information about synthesis conditions and conclusion form the presented research.

Table S1. Examples of synthesis method and properties of upconverting SrF_2 from literature.

Material	Size (nm)	Novelty	Synthesis method	Ref.
SrF_2:Yb^{3+}, Er^{3+}	5 - 25	nanocrystals with different size due to reaction time	solvothermal in a mixture of water/ethanol/ oleic acid/sodium oleate, T= 180°C, t= 4/16/72 h	1
SrF_2:Yb^{3+}, Tm^{3+}	8	influence of Na^+ and K^+ on spectroscopic properties of SrF_2:Yb^{3+}, Tm^{3+} in water and D_2O	hydrothermal synthesis, sodium citrate, T=190 °C, t= 6 h, 2.5× NH_4F	2
SrF_2:Nd^{3+}	10	NPs for deep tissue, autofluorescence free and high resolution in vivo imaging using emission band at 1.340 μ, toxicological research	hydrothermal synthesis with potassium citrate, T=190 °C, t= 6 h, 2.5× NH_4F	3
SrF_2:Yb^{3+}, Tm^{3+}	9	intense emission in UV of water colloid, correlation between the temperature and upconversion emission intensities in a D2O colloidal dispersion at different temperatures (10°C and 60°C)	hydrothermal synthesis, sodium citrate, T=190 °C, t= 6 h, 1.5× NH_4F	4
SrF_2:Yb^{3+}, Tm^{3+} @CaF_2:Gd^{3+}	20	first synthesis of core–shell NPs via a two-step hydrothermal method, magnetic characterization of core–shell SrF_2:Yb^{3+}, Tm^{3+} @CaF_2:Gd^{3+}	two-step hydrothermal method, with sodium citrate,1st step T=190 °C , t= 6 h, 2nd step T=200°C , t= 6 h	5
SrF_2:Yb^{3+}, Tm^{3+} @SrF_2:Yb^{3+}, Er^{3+}	10-12	investigation of interactions between Ln-doped fluoride NPs and biomolecules (ubiquitin)	two step hydrothermal synthesis, sodium citrate, T=190 °C , t= 3 h, 2.5× NH_4F (1st and 2nd step)	6
SrF_2:Yb^{3+}, Er^{3+}	8-41	developed the method to predict the temperature calibration curve of any upconverting thermometer, independently of the	hydrothermal synthesis, sodium citrate, T=190 °C, t= 6 h	7
SrF_2:Nd^{3+} SrF_2:Nd^{3+}, Gd^{3+}	6.5-9	high thermal relative sensitivity in range 20 C - 65 C,	hydrothermal synthesis, potassium citrate, T=190 °C, t= 3 h or 10 min	8
SrF_2:Yb^{3+}, Er^{3+}	100-300	quantum yield of upconversion SrF_2, with different amount of Yb^{3+} ions	precipitation with further calcination in 600°C	9
SrF_2:Eu^{3+}	10-40	investigation of symmetry site of Eu^{3+} in cubic SrF_2, achieving long Eu^{3+} lifetime	hydrothermal method with sodium/potassium citrate, T=190 °C , t= 10, 35, 360, and 480 min, 2.5×	10
SrF_2:Yb^{3+},Tm^{3+}@ Y^{3+} @Yb^{3+},Er^{3+}, Nd^{3+} @Nd^{3+}	8.5-26.6	optimization of thermal relative sensitivity by changing Er^{3+} content in 20–50 °C temperature range, measured in water colloid, possibility of a multicolour UC emission	four sequential hydrothermal reaction steps with potassium citrate, 2.5× NH_4F, T=190 °C , t= 3 h for each step	11
SrF_2:Yb^{3+}, Er^{3+}	10/ 41	calculation of photothermal conversion efficiencies, investigation of photon-to-heat conversion efficiency	hydrothermal reaction with sodium citrate, T=190 °C, t= 6 h	12
SrF_2:Yb^{3+}, Er^{3+}	-	investigation of upconversion luminescence of Er^{3+} ions for a concentration series of phosphors upon excitation by laser radiation in the region of 1.5 µm	co-precipitation from aqueous solutions	13
SrF_2:Yb^{3+}, Ho^{3+}	39	investigation of magnetization values and abnormal thermal of band at 656 nm	hydrothermal method, with EDTA and NH_4BF_4 T=200 °C, t= 5 h, pH 5	14

Structure and morphology

Table S2. Size of obtained NPs, calculated from the Scherrer equation on the basis of XRD analysis[15].

Co-reagent	Reaction time (h)	Size (nm)					
		SrF_2:20%Yb^{3+},1%Ho^{3+}		SrF_2:20%Yb^{3+},1%Er^{3+}		SrF_2:20%Yb^{3+},0.25 %Tm^{3+}	
		1.5× NH_4F	3× NH_4F	1.5× NH_4F	3× NH_4F	1.5× NH_4F	3× NH_4F
NaCit	6	11.7 ± 0.1		11.8 ± 0.2		11.6 ± 0.2	
	12	12.8 ± 0.1	28.1 ± 0.3	13.5 ± 0.4	38.5 ± 0.4	11.5 ± 0.1	38.9 ± 0.4
NH_4Cit	6	12.6 ± 0.1		14.7 ± 0.5		14.1 ± 0.3	
	12	14.2 ± 0.3	36.2 ± 0.9	15.1 ± 0.5	38.8 ± 0.7	15.3 ± 0.2	36.6 ± 0.7

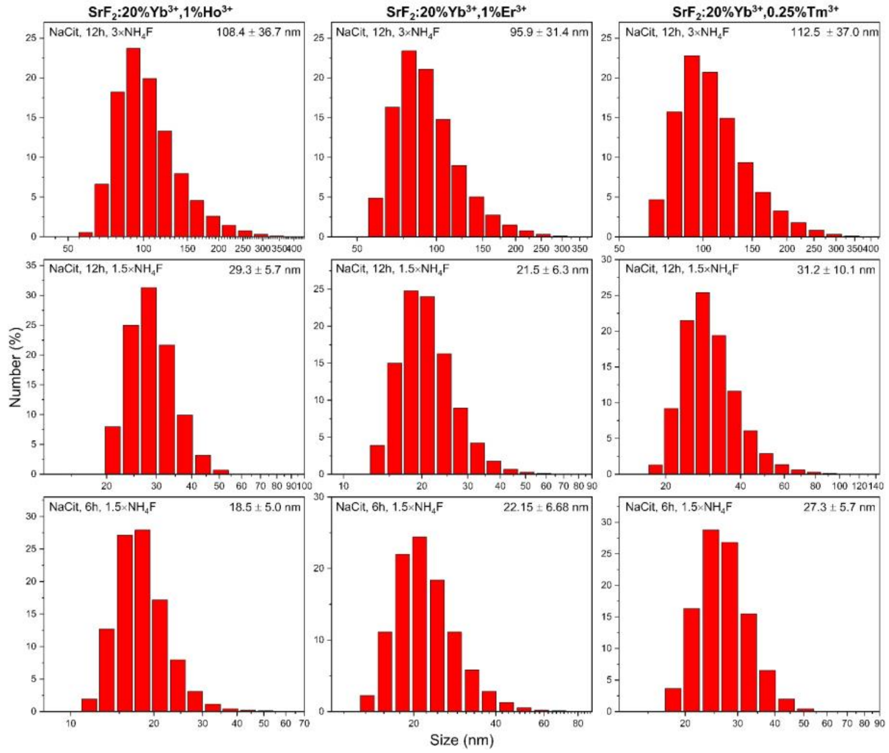

Fig. S1. Hydrodynamic diameters obtained by DLS analysis of the synthesised SrF_2:Yb^{3+},Ln^{3+} NPs in the presence of NaCit as a co-reagent.

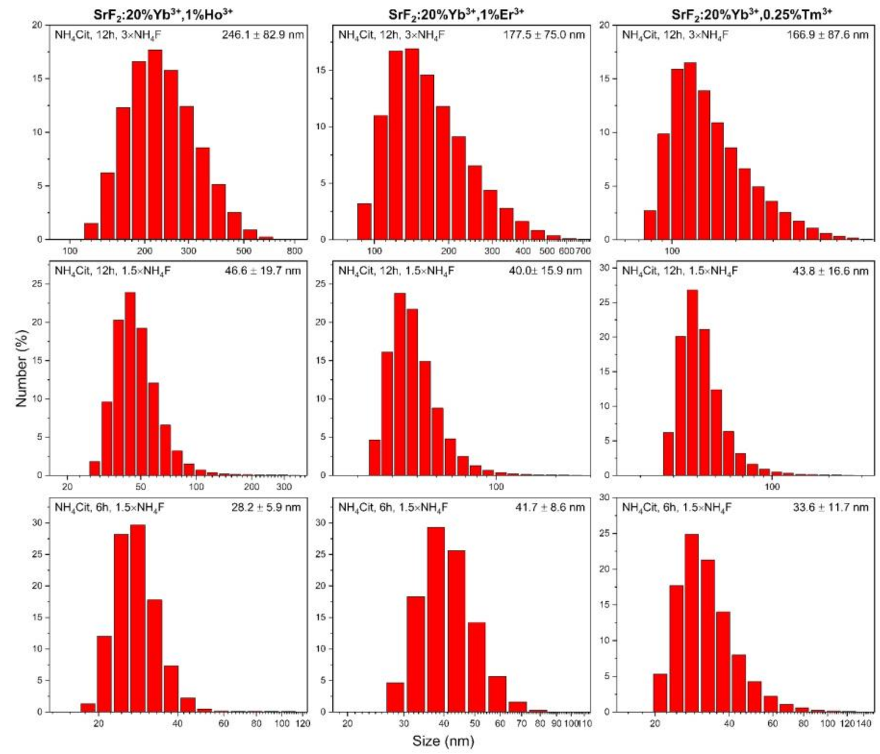

Fig. S2. Hydrodynamic diameters obtained by DLS analysis of the synthesised SrF_2:Yb^{3+},Ln^{3+} NPs in the presence of NH_4Cit as a co-reagent.

Table S3. Metal ions composition of synthesised NPs analysed by ICP-OES (uncertainty of ICP-OES analysis was below ± 5% of the value).

**Co-reagent**	**Reaction time (h)**	**Dopants**	**Molar percentage (%)**					
			**1.5× NH_4F**			**3× NH_4F**		
			**Sr^{2+}**	**Yb^{3+}**	**Ln^{3+}**	**Sr^{2+}**	**Yb^{3+}**	**Ln^{3+}**
**NaCit**	**6**	**20%Yb^{3+},1%Ho^{3+}**	75.42	23.46	1.12			
		**20%Yb^{3+},1%Er^{3+}**	77.79	21.39	0.82			
		**20%Yb^{3+},0.25%Tm^{3+}**	76.97	22.71	0.32			
	**12**	**20%Yb^{3+},1%Ho^{3+}**	74.51	24.17	1.32	74.61	24.02	1.37
		**20%Yb^{3+},1%Er^{3+}**	77.40	20.97	1.63	75.22	23.63	1.15
		**20%Yb^{3+},0.25%Tm^{3+}**	75.43	24.34	0.23	75.54	24.24	0.22
**NH_4Cit**	**6**	**20%Yb^{3+},1%Ho^{3+}**	75.63	23.07	1.30			
		**20%Yb^{3+},1%Er^{3+}**	75.95	22.76	1.29			
		**20%Yb^{3+},0.25%Tm^{3+}**	76.90	22.76	0.34			
	**12**	**20%Yb^{3+},1%Ho^{3+}**	75.16	22.99	1.85	70.43	28.26	1.31
		**20%Yb^{3+},1%Er^{3+}**	74.52	23.42	2.07	70.70	28.29	1.00
		**20%Yb^{3+},0.25%Tm^{3+}**	76.66	22.98	0.36	71.28	28.51	0.22

Table S4. Results of elemental analysis of chosen samples (doped with 20%Yb^{3+},1%Er^{3+}).

Synthesis conditions	N (wt%)	C (wt%)	H (wt%)
**NH_4Cit, 12h, 1.5x NH_4F**	0.246	1.935	0.296
	0.270	1.950	0.299
**NH_4Cit, 12h, 3x NH_4F**	0.106	0.529	0.387
	0.112	0.535	0.090
**NaCit, 12h, 3x NH_4F**	0.020	0.645	0.116
	0.030	0.526	0.098

Table S5. Cell parameter for cubic structure SrF_2:Yb^{3+},Ln^{3+} calculated in Maud software[16].

Co-reagent	NH_4F excess	Reaction time (h)	SrF_2:20%Yb^{3+},1%Ho^{3+}		SrF_2:20%Yb^{3+},1%Er^{3+}		SrF_2:20%Yb^{3+},0.25 %Tm^{3+}	
			a **(Å)**	***V*** **(Å^3)**	***a*** **[Å]**	***V*** **(Å^3)**	***a*** **[Å]**	***V*** **(Å^3)**
**NaCit**	**1.5×**	**6**	5.73(8)	188.92(2)	5.73(8)	188.94(8)	5.73(7)	188.86(1)
		**12**	5.73(7)	188.86(9)	5.73(7)	188.86(9)	5.73(8)	188.89(7)
	**3×**	**12**	5.73(2)	188.31(1)	5.73(3)	188.39(1)	5.73(4)	188.48(9)
**NH_4Cit**	**1.5×**	**6**	5.73(4)	188.56(9)	5.73(6)	188.71(7)	5.73(7)	188.78(1)
		**12**	5.73(7)	188.82(4)	5.73(7)	188.85(7)	5.73(8)	188.90(6)
	**3×**	**12**	5.73(0)	188.09(4)	5.73(0)	188.10(5)	5.73(0)	188.16(5)
Reference pattern SrF_2 ICSD #40414, a = 5.794 Å, V = 194.50(7) Å^3								

Table S6. Hydrodynamic diameters of obtained NPs determined by DLS measurements.

Co-reagent	Reaction time (h)	Hydrodynamic diameter (nm)					
		SrF_2:20%Yb^{3+},1 %Ho^{3+}		SrF_2:20%Yb^{3+},1%Er^{3+}		SrF_2:20%Yb^{3+},0.25%Tm^{3+}	
		1.5× NH_4F	3× NH_4F	1.5× NH_4F	3× NH_4F	1.5× NH_4F	3× NH_4F
**NaCit**	**6**	18.5± 5.0		22.15 ± 6.68		27.3±5.7	
	**12**	29.3± 5.7	108.4± 36.7	21.5± 6.3	95.9± 31.4	31.2± 10.1	112.5± 37.0
**NH_4Cit**	**6**	28.2± 5.9		41.7± 8.6		33.6± 11.7	
	**12**	46.6± 19.7	246.1± 82.9	40.0± 15.9	177.5± 75.0	43.8± 16.6	166.9± 87.6

Table S7. Summary of zeta potential measurements for obtained SrF_2:Yb^{3+},Ln^{3+} particles for physiological pH value.

Co-reagent	Reaction time (h)	SrF_2:20%Yb^{3+},1%Ho^{3+}				SrF_2:20%Yb^{3+},1%Er^{3+}				SrF_2:20%Yb^{3+},0.25%Tm^{3+}			
		1.5× NH_4F		3× NH_4F		1.5× NH_4F		3× NH_4F		1.5× NH_4F		3× NH_4F	
		pH	Zeta potential (mV)	pH	Zeta potential (mV)	pH	Zeta potentia l (mV)	pH	Zeta potential (mV)	pH	Zeta potential (mV)	pH	Zeta potential (mV)
NaCit	6	7.36	-22.7 ± 8.5			7.44	-23.3 ±8.38			7.34	-20.1 ± 3.7		
	12	7.37	-14.9 ± 5.4	7.40	-33.3 ± 4.4	7.40	-21.0 ± 4.2	7.46	-19.5 ± 8.1	7.52	-27.9 ± 5.8	7.32	-29.8 ± 4.2
NH_4Cit	6	7.33	-28.3 ± 4.2			7.41	-24.7 ± 4.5			7.34	-23.3 ± 4.9		
	12	7.32	-27.8 ± 3.8	7.43	-27.9 ± 4.9	7.45	-20.1 ± 3.8	7.37	-29.6 ± 3.8	7.44	-27.7 ± 4.5	7.34	-26.0 ± 3.4

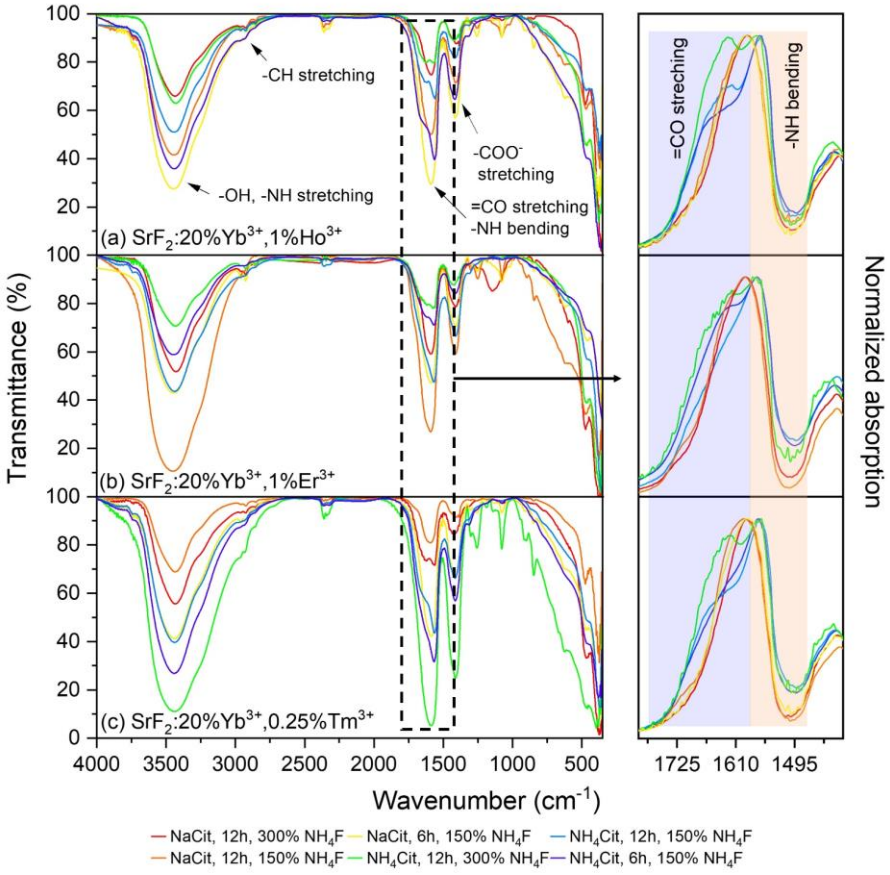

Fig. S3. Fourier transformed infrared spectroscopy (FT-IR) spectra of the obtained samples (left) and normalized absorption of samples in the 1400-1800 cm^{-1} range (right). Absorption of samples obtained in the presence pf NH_4Cit presented small shift to shorter wavenumber as the result of higher amount of NH_4^+ ions.

Luminescence properties

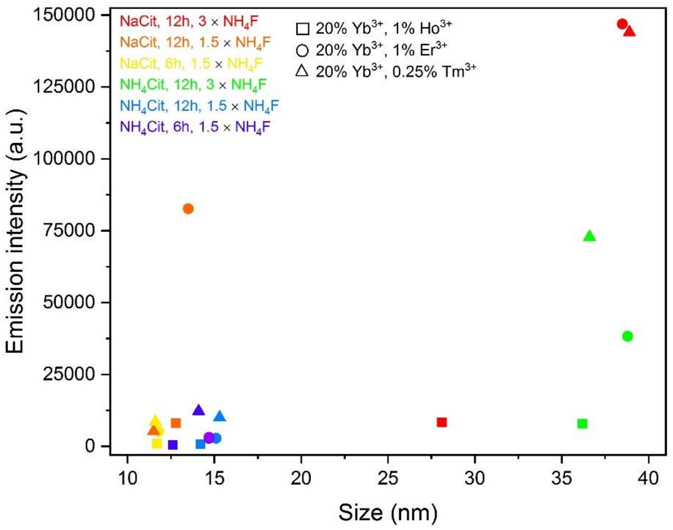

Fig. S4. Relationships between the integral emission intensity and NPs size of $SrF_2:Yb^{3+},Ln^{3+}$. Calculations based on the emission spectra, measured under λ_{ex} = 976 nm pulsed excitation source (at 15 mJ·cm^{-2}).

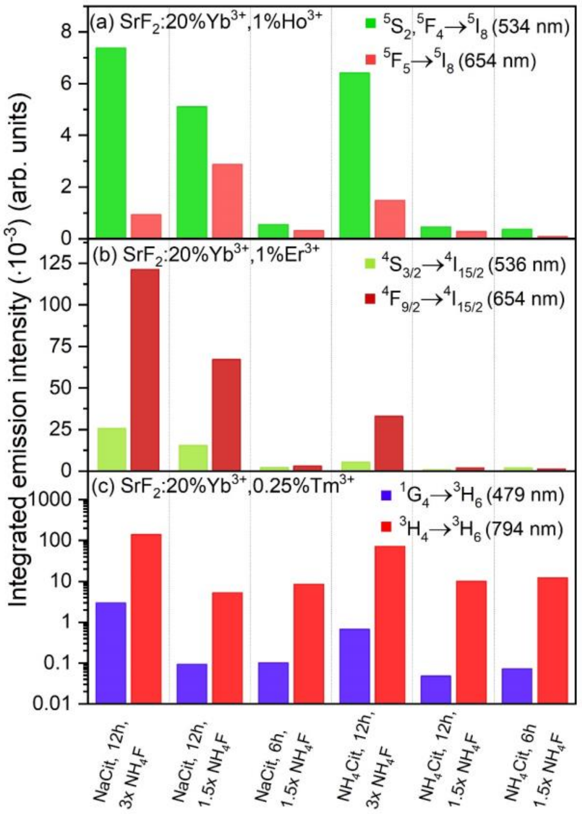

Fig. S5. Integrated luminescence intensities of SrF_2:20%Yb^{3+},x%Ln^{3+} samples: a) 20%Yb^{3+},1%Ho^{3+}, b) 20%Yb^{3+},1%Er^{3+}, c) 20%Yb^{3+},0.25%Tm^{3+}, calculated from the spectra measured under λ_{ex} = 976 nm pulsed excitation source (at 15 mJ·cm^{-2}).

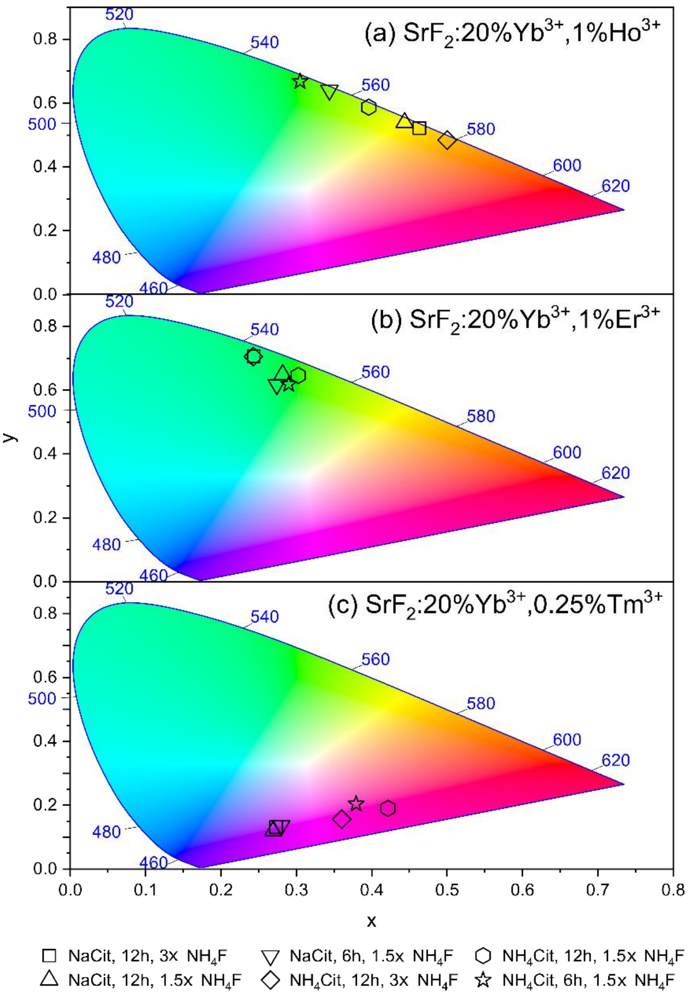

Fig. S6. CIE chromaticity diagrams of SrF_2:Yb^{3+},Ln^{3+} NPs, taken from the emission spectra measured under λ_{ex} = 976 nm pulsed excitation source (at 15 mJ·cm^{-2}).

Because of the non-exponential character of luminescence decays, the following equation was used for lifetimes calculation:

$$\tau = \frac{\int_0^\infty tI(t)dt}{\int_0^\infty I(t)dt}$$

where *τ* is the decay time, and *I(t)* is the intensity at time *t*.[17]

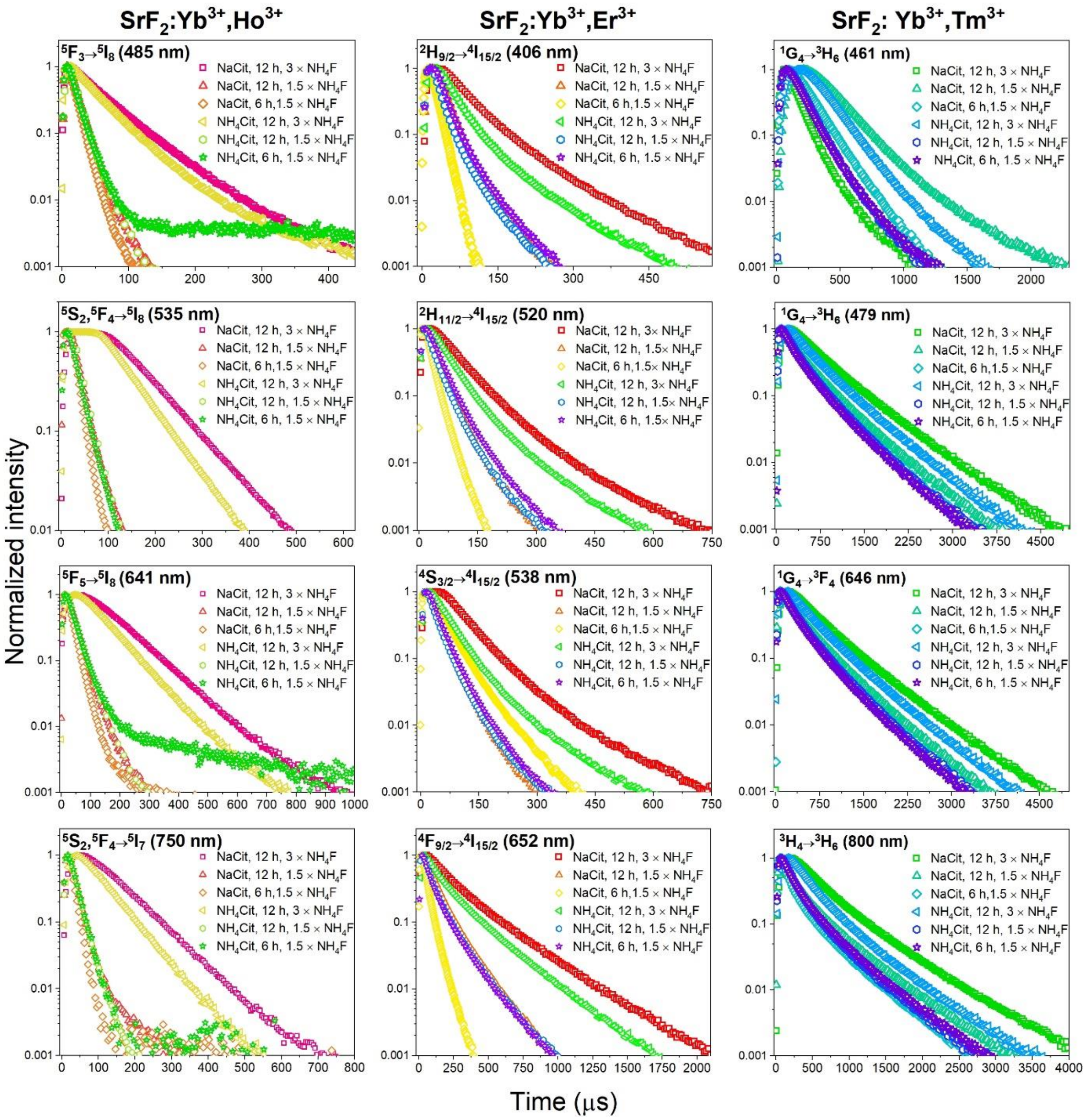

Fig. S7. Emission decays of SrF_2:Yb^{3+},Ln^{3+} NPs measured under λ_{ex} = 976 nm pulsed excitation source (at 15 mJ·cm^{-2}).

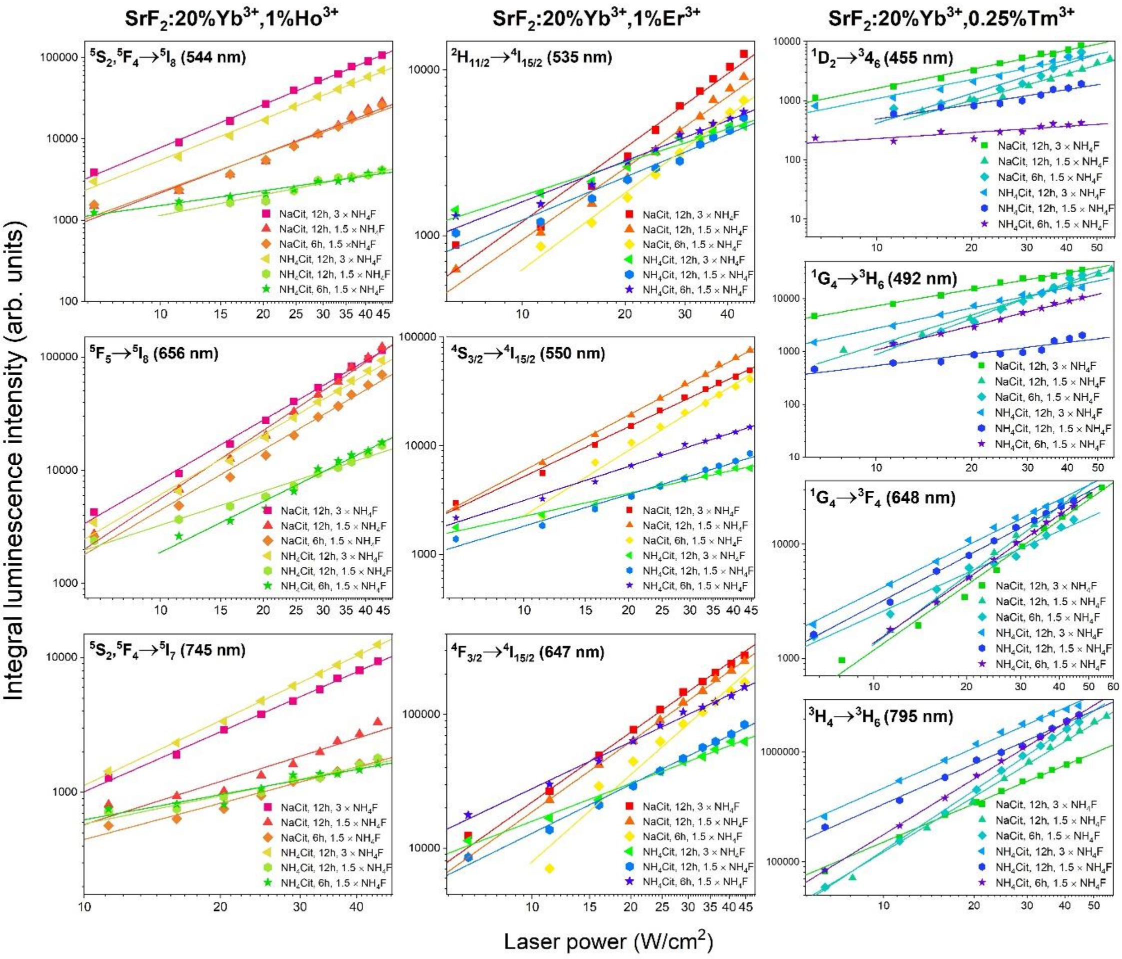

Fig. S8. Double-logarithmic plots of the upconversion emission intensity versus laser power density (λ_{ex} = 976 nm), for $SrF_2:Yb^{3+},Ln^{3+}$ NPs.

Biological properties

Cytotoxicity assays

For the cytotoxicity test, $5{\cdot}10^3$ cells/well were seeded at 96-well plate and incubated for 24 h. Afterwards, 50 µl of several different concentrations of the NPs diluted in PBS were added to 150 µl of culture medium in the particular wells resulting in a final concentration of 100, 50, 25, 12.5 and 6.25 µg/mL, and the cells were further incubated for 48 h. Phosphate buffered saline (PBS, Sigma-Aldrich) was used as a control.

The influence of the studied NPs on the cells was investigated by cell proliferation WST-1 assay (Takarra) according to literature.[18] This colorimetric assay is based on the cleavage of tetrazolium salts by mitochondrial dehydrogenase in viable cells. After 48 h of incubation with the NPs, WST-1 Cell Proliferation Reagent was added (10 µL per each well) and the cells were incubated again for 4 h. Then, 100 µL of supernatant was transferred to fresh wells to avoid absorption of light by the NPs

and absorbance at 450 nm was measured using multiwell plate reader (Zenyth, Biochrom). The reference wavelength was 620 nm. The cell viability was expressed as the respiration activity normalised to untreated cells.

The fluorescent Live/Dead cell viability assay allows distinction of live cells with intact plasma membranes from dead cells with compromised membranes. In this test, the cells were seeded in black polystyrene 96-wells flat bottom plate with the transparent bottom (Greiner Bio-One GmbH). Following 48 h exposure to the NPs, the cells were incubated with 2 μM calcein AM, 2 μM ethidium homodimer-1 and 8 μM Hoechst 33342 (ThermoFisher Scientific) containing DPBS (100 μL/well) for 30 minutes at 37 °C. Finally, the cells were analysed with the IN Cell Analyzer 2000 (GE Healthcare Life Sciences). Viable cells were imaged using the FITC/FITC excitation/emission filters while for the dead cells the TexasRed/TexasRed ex/em filter combination was applied. DAPI/DAPI was applied to detect the Hoechst 33342 blue signal. A minimum of 20 fields was imaged per well with a 20× magnification. Analysis of the collected images was performed with the IN Cell Developer Toolbox software (GE Healthcare Life Sciences) using an in-house developed protocol. At first, the total cell number was retrieved from the DAPI images by means of defining and counting the nuclei. Subsequently, the number of viable cells from the FITC images and the number of dead cells from the TexasRed images were determined.

Each experiment was repeated three times and all of the samples in one experiment were tested in triplets. The results were analysed using the non-parametric Kruskal-Wallis test with the Statistica 13 software package. Differences were considered statistically significant at $p < 0.05$. The data are shown as mean values with the standard deviation (means ± SD).

Cellular uptake of nanoparticles

To image the cells treated with the NPs, $1.25 \cdot 10^4$ cells/well were seeded at Lab-Tek™ chamber slides which consist of removable polystyrene media chambers attached to standard glass slides and grown for 24 h at 37 °C in a humidified atmosphere supplemented with 5% CO_2. Afterwards, 50 μl of NPs (100 μg/mL in PBS) was added to the cells which were further incubated for 24 h. Next, the cells were fixed with 4% formaldehyde solution (Sigma-Aldrich) and stained with concanavalin A (Alexa Fluor 647 Conjugate, ThermoFisher Scientific) and Hoechst 33342. Afterwards, the samples were imaged using confocal laser scanning microscopy (CLSM, Zeiss, LSM 780) equipped with a tuneable infrared laser. The fluorescence emission was detected using two channels: the spectrum of luminescence under infrared excitation (an up-conversion process made by NPs) and the spectrum

of fluorescence under VIS and UV excitation (Hoechst 33342 and concanavalin A). The above procedure was applied to make sure that the detection of some compounds inside the cells came from the up-converting NPs.

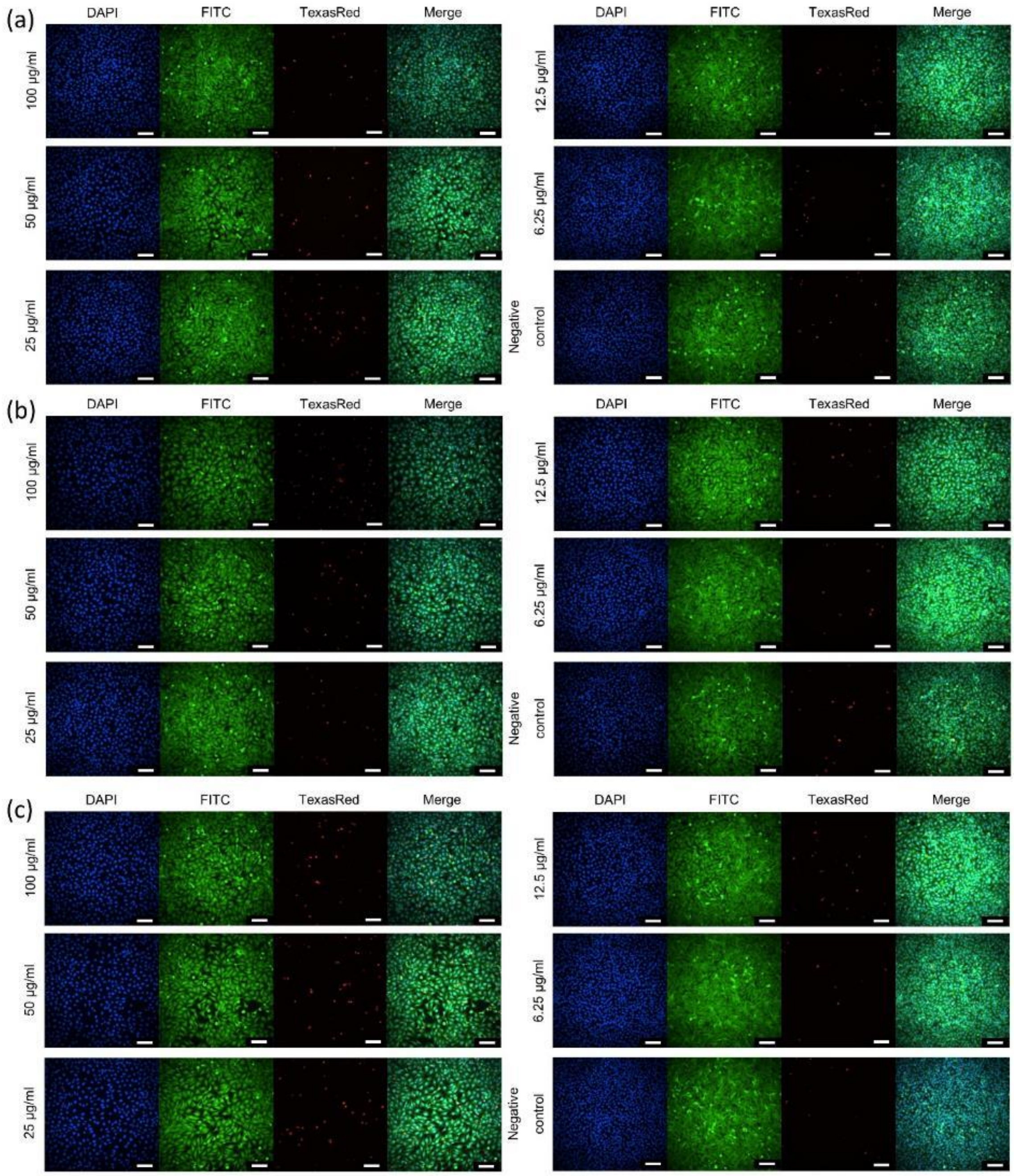

Fig. S9. Representative high-content images of MSU1.1 cells exposed to (a) SrF_2:20%Yb^{3+},1%Ho^{3+}, (b) SrF_2:20%Yb^{3+},1%Ho^{3+}@PEG(COOH)$_2$, (c) SrF_2:20%Yb^{3+},1%Ho^{3+}@PAA NPs (6.25 – 100 µg/mL). The images were obtained using different filters to detect the nuclei (DAPI), live cells (FITC), and dead cells (TexasRed). The scale bars denote 100 µm.

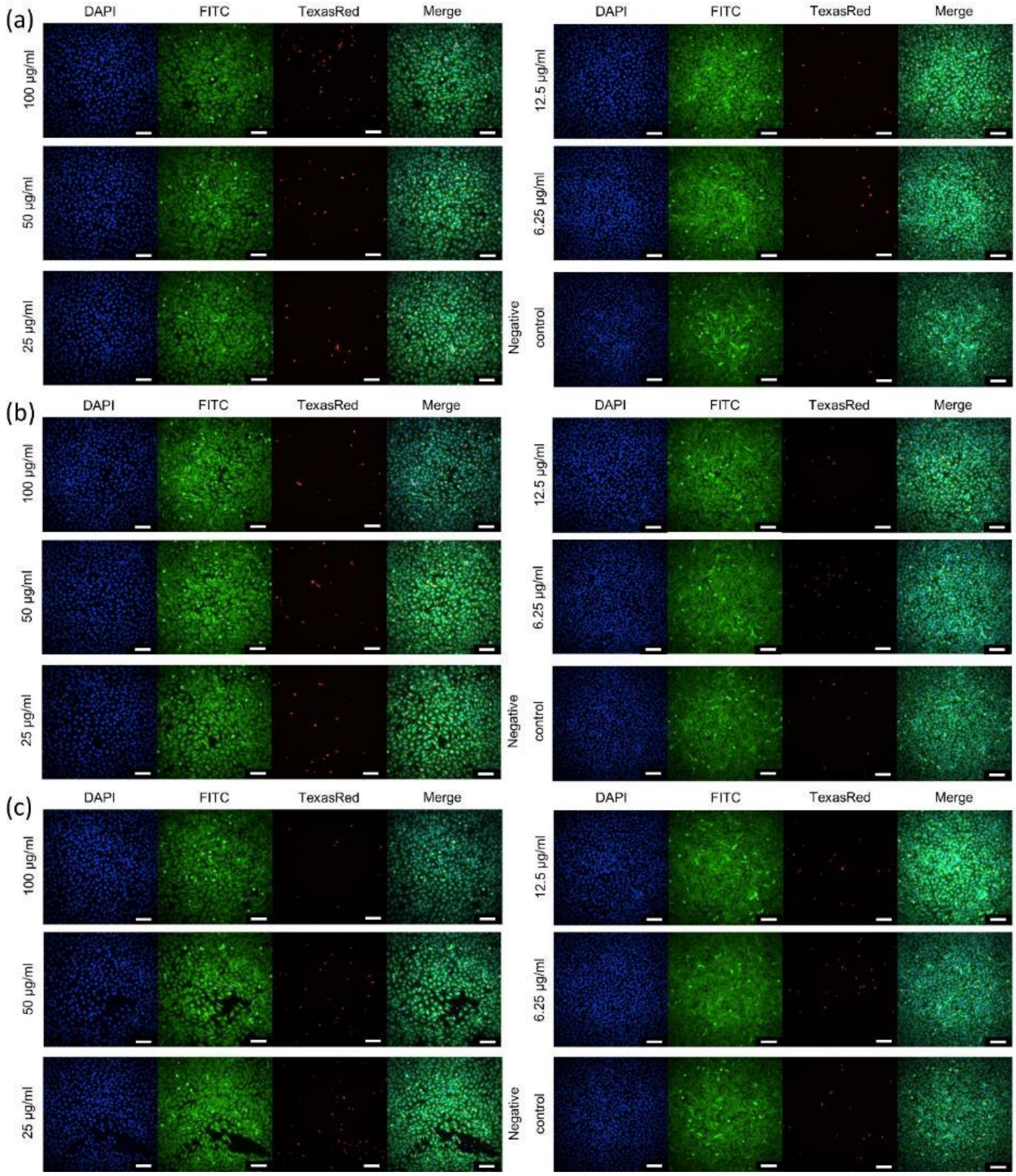

Fig. S10. Representative high-content images of MSU1.1 cells exposed to (a) SrF_2:20%Yb^{3+},1%Er^{3+}, (b) SrF_2:20%Yb^{3+},1%Er^{3+}@$(COOH)_2$, (c) SrF_2:20%Yb^{3+},1%Er^{3+}@PAA NPs (6.25 – 100 µg/mL). The images were obtained using different filters to detect the nuclei (DAPI), live cells (FITC), and dead cells (TexasRed). The scale bars denote 100 µm.

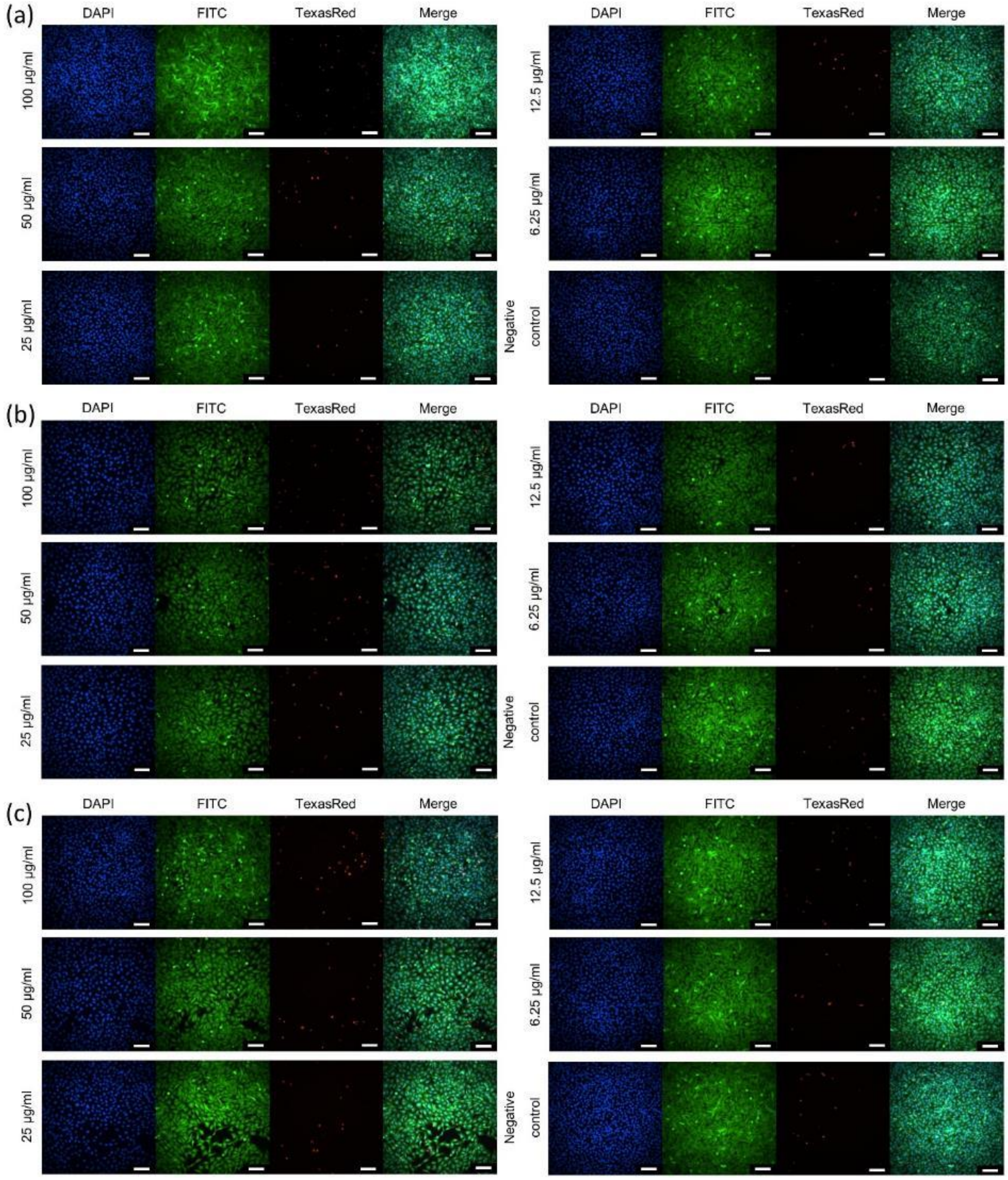

Fig. S11. Representative high-content images of MSU1.1 cells exposed to (a) SrF_2:20%Yb^{3+},0.25%Tm^{3+}, (b) SrF_2:20%Yb^{3+},0.25%Tm^{3+}@$(COOH)_2$, (c) SrF_2:20%Yb^{3+},0.25%Tm^{3+}@PAA NPs (6.25 – 100 µg/mL). The images were obtained using different filters to detect the nuclei (DAPI), live cells (FITC), and dead cells (TexasRed). The scale bars denote 100 µm.

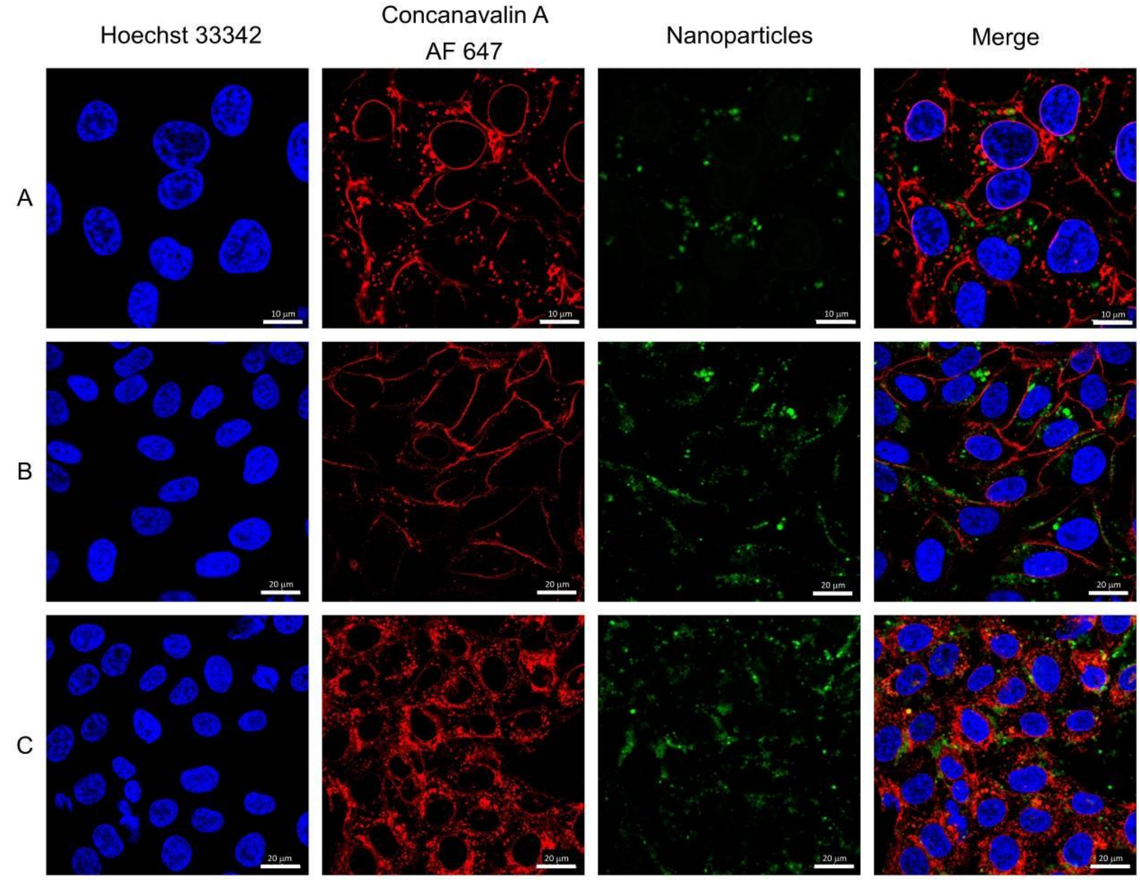

Fig. S12. Human fibroblasts after 24h incubation with (A) SrF_2:20%Yb^{3+},1%Ho^{3+}, (B) SrF_2:20%Yb^{3+},1%Ho^{3+}@$(COOH)_2$, (C) SrF_2:20%Yb^{3+},1%Ho^{3+}@PAA imaged using confocal laser scanning microscopy equipped with a tuneable infrared laser. Red colour - cell membrane (concanavalin 647, exc. 633 nm), blue colour – cell nuclei (DAPI, exc. 405 nm), green colour – NPs (NPs' luminescence, infrared excitation).

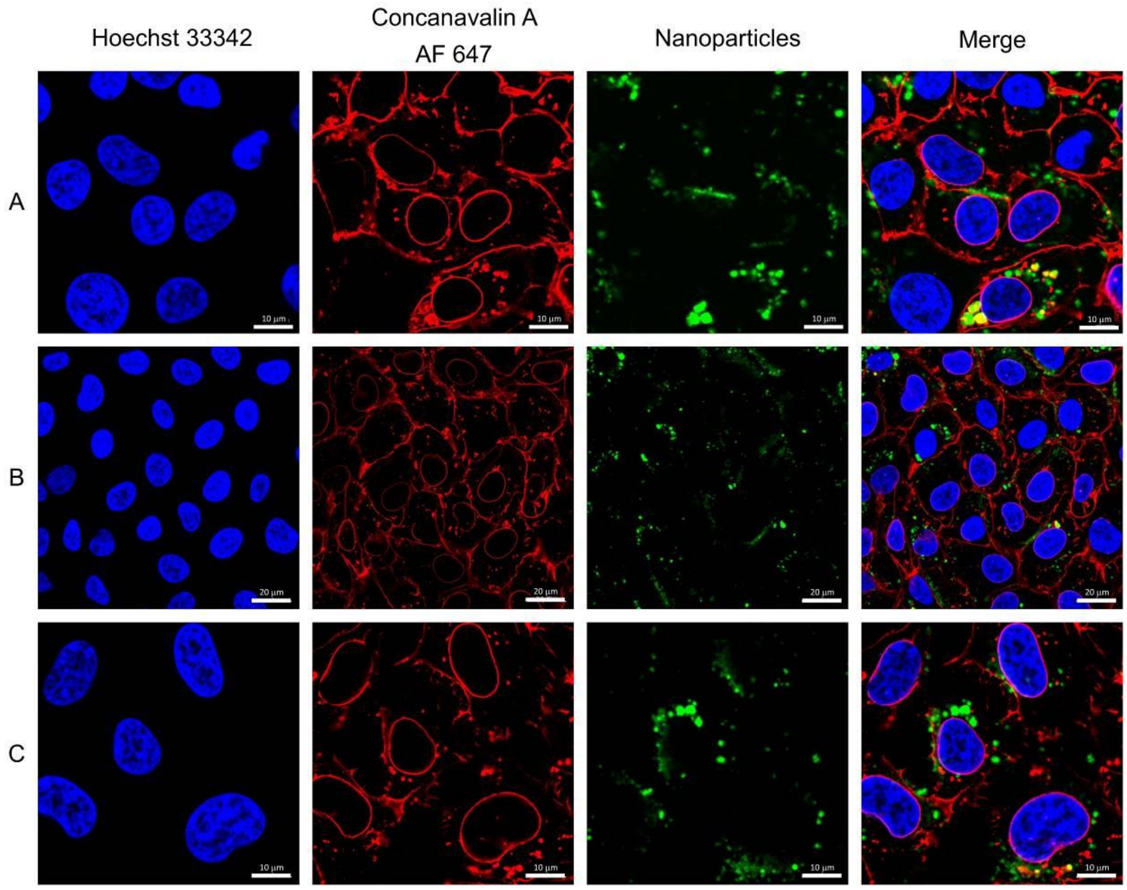

Fig. S13. Human fibroblasts after 24h incubation with (A) SrF_2:20%Yb^{3+},1%Er^{3+}, (B) SrF_2:20%Yb^{3+},1%Er^{3+}@$(COOH)_2$, (C) SrF_2:20%Yb^{3+},1%Er^{3+}@PAA, imaged using confocal laser scanning microscopy equipped with a tuneable infrared laser. Red colour - cell membrane (concanavalin 647, exc. 633 nm), blue colour – cell nuclei (DAPI, exc. 405 nm), green colour – NPs (NPs' luminescence, infrared excitation).

References

1. Sun, J., Xian, J. & Du, H. Facile synthesis of well-dispersed SrF_2:Yb^{3+}/Er^{3+} upconversion nanocrystals in oleate complex systems. *Appl. Surf. Sci.* **257,** 3592–3595 (2011).

2. Pedroni, M. *et al.* Water (H_2O and D_2O) Dispersible NIR-to-NIR Upconverting Yb^{3+}/Tm^{3+} Doped MF_2 (M = Ca, Sr) Colloids: Influence of the Host Crystal. *Cryst. Growth Des.* **13,** 4906–4913 (2013).

3. Villa, I. *et al.* 1.3 µm emitting SrF_2:Nd^{3+} nanoparticles for high contrast in vivo imaging in the second biological window. *Nano Res.* **8,** 649–665 (2015).

4. Quintanilla, M., Cantarelli, I. X., Pedroni, M., Speghini, A. & Vetrone, F. Intense ultraviolet upconversion in water dispersible SrF_2:Tm^{3+},Yb^{3+} nanoparticles: the effect of the environment on light emissions. *J. Mater. Chem. C* **3,** 3108–3113 (2015).

5. Li, A.-H. *et al.* Upconversion-luminescent/magnetic dual-functional sub-20 nm core–shell

SrF_2:Yb,Tm@CaF_2:Gd heteronanoparticles. *Dalt. Trans.* **45,** 5800–5807 (2016).

6. Zanzoni, S., Pedroni, M., D'Onofrio, M., Speghini, A. & Assfalg, M. Paramagnetic Nanoparticles Leave Their Mark on Nuclear Spins of Transiently Adsorbed Proteins. *J. Am. Chem. Soc.* **138,** 72–75 (2016).

7. Balabhadra, S., Debasu, M. L., Brites, C. D. S., Ferreira, R. A. S. & Carlos, L. D. Upconverting Nanoparticles Working As Primary Thermometers In Different Media. *J. Phys. Chem. C* **121,** 13962–13968 (2017).

8. Pedroni, M. *et al.* Colloidal nanothermometers based on neodymium doped alkaline-earth fluorides in the first and second biological windows. *Sensors Actuators, B Chem.* **250,** 147–155 (2017).

9. Kuznetsov, S. *et al.* Up-conversion quantum yields of SrF_2:Yb^{3+},Er^{3+} sub-micron particles prepared by precipitation from aqueous solution. *J. Mater. Chem. C* **6,** 598–604 (2018).

10. Cortelletti, P. *et al.* Luminescence of Eu^{3+} Activated CaF_2 and SrF_2 Nanoparticles: Effect of the Particle Size and Codoping with Alkaline Ions. *Cryst. Growth Des.* **18,** 686–694 (2018).

11. Cortelletti, P. *et al.* Tuning the sensitivity of lanthanide-activated NIR nanothermometers in the biological windows. *Nanoscale* **10,** 2568–2576 (2018).

12. Balabhadra, S., Debasu, M. L., Brites, C. D. S., Ferreira, R. A. S. & Carlos, L. D. Radiation-to-heat conversion efficiency in SrF_2:Yb^{3+}/Er^{3+} upconverting nanoparticles. *Opt. Mater.* **83,** 1–6 (2018).

13. Lyapin, A. A. *et al.* Upconversion Luminescence of Fluoride Phosphors SrF_2:Er,Yb under Laser Excitation at 1.5 µm. *Opt. Spectrosc.* **125,** 537–542 (2018).

14. Du, S. & Wang, Y. A broad-range temperature sensor dependent on the magnetic and optical properties of SrF_2:Yb^{3+},Ho^{3+}. *CrystEngComm* **21,** 1452–1457 (2019).

15. Scherrer, P. Bestimmung der Grösse und der inneren Struktur von Kolloidteilchen mittels Röntgenstrahlen. *Nachr. Ges. Wiss. Göttingen* **26,** 98–100 (1918).

16. Lutterotti, L. & Bortolotti, M. Object oriented programming and fast computation techniques in Maud, a program for powder diffraction analysis written in java. *Compcomm. Newsl.* **1,** 43–50 (2003).

17. Lakowicz, J. R. *Principles of Fluorescence Spectroscopy*. (Springer, 2006).

18. Woźniak, A. *et al.* Size and shape-dependent cytotoxicity profile of gold nanoparticles for biomedical applications. *J. Mater. Sci. Mater. Med.* **28,** 92 (2017).
